# Supplementary material for: Extraction, phytochemical characterization and anti-cancer mechanism of Haritaki churna: An ayurvedic formulation
Source: PLoS One. 2023 May 31;18(5):e0286274. doi: 10.1371/journal.pone.0286274 (PMC10231837; doi:10.1371/journal.pone.0286274)
Supplement: S1 Data — (DOCX) [file pone.0286274.s001.docx]

**Supplementary data-S1_ NMR and LC-MS data for compounds isoalted from HCAE**

Figure S1: Yield of reverse phase open column chromatography fractions of aqueous extract of haritaki churna

Figure S2: 1H NMR analysis of compound 1

Figure S3: Mass spectra analysis of compound 1

Figure S4: 1H NMR analysis of compound 2

Figure S5: Mass spectra analysis of compound 2

Figure S6: 1H NMR analysis of compound 3

Figure S7: Mass spectra analysis of compound 3

Figure S8: 1H NMR analysis of compound 4

Figure S9: Mass spectra analysis of compound 4

Figure S10: 1H NMR analysis of compound 5

Figure S11: Mass spectra analysis of compound 5

Figure S12: 1H NMR analysis of compound 6 (Acetone d6)

Figure S13: 1H NMR analysis of compound 6 (Acetone d6 + D2O)

Figure S14: Mass spectra analysis of compound 6

Figure S15: 1H NMR analysis of compound 7

Figure S16: Mass spectra analysis of compound 7

Figure S17: 1H NMR analysis of compound 8

Figure S18: Mass spectra analysis of compound 8

Figure S19: 1H NMR analysis of compound 9

Figure S20: Mass spectra analysis of compound 9

Figure S21: 1H NMR analysis of compound 10

Figure S22: Mass spectra analysis of compound 10

Figure S23: 1H NMR analysis of compound 11

Figure S24: Mass spectra analysis of compound 11

Figure S25: 1H NMR analysis of compound 12

Figure S26: Mass spectra analysis of compound 12

Figure S27: 1H NMR analysis of compound 13

Figure S28: Mass spectra analysis of compound 13

**Figure S1: Yield of reverse phase open column chromatography fractions of aqueous extract of haritaki churna**

The above graph represents the yield of fractions from open column chromatography. The parental fraction represent the yield from 1 gram of Haritaki churna when extracted in water. The fraction 1 is 0% MeOH fraction, fraction 2 is 20% MeOH, fraction 3 is 50% MeOH, fraction 4 is 80% MeOH, fraction 5 is 100% MeOH and the last fraction is the loss accounted.

**Compound 1**

**
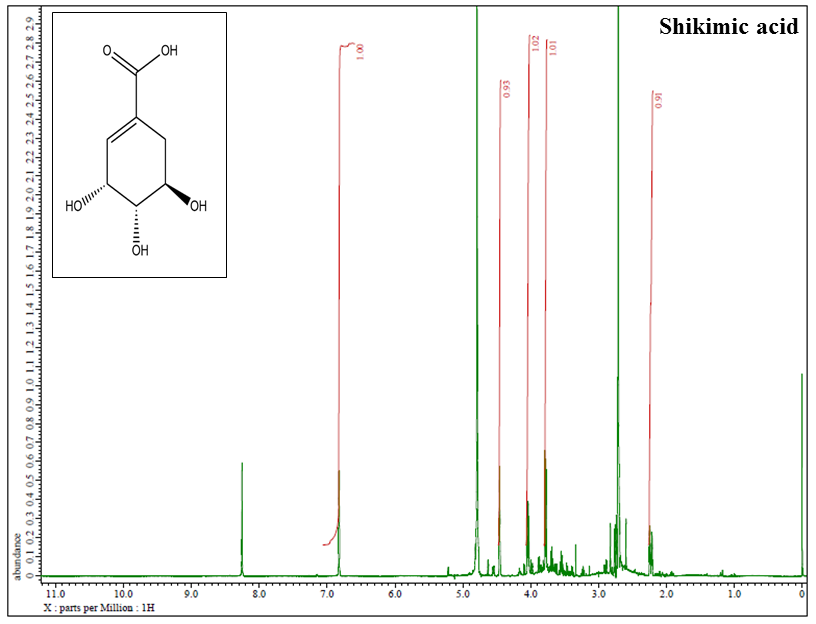
**

**Figure S2: 1H NMR analysis of compound 1**

**
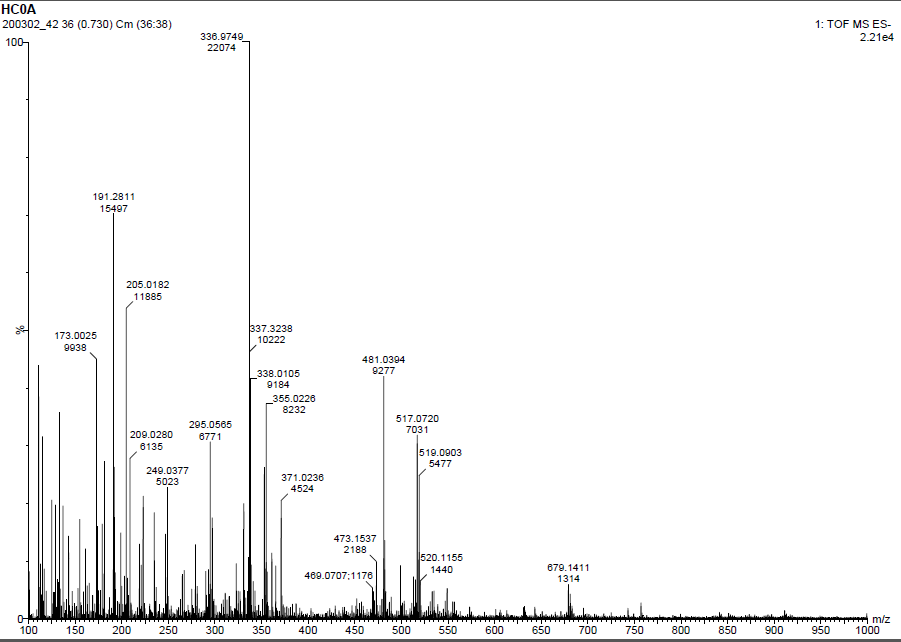
**

**Figure S3: Mass spectra analysis of compound 1**

**Compound 2**

**
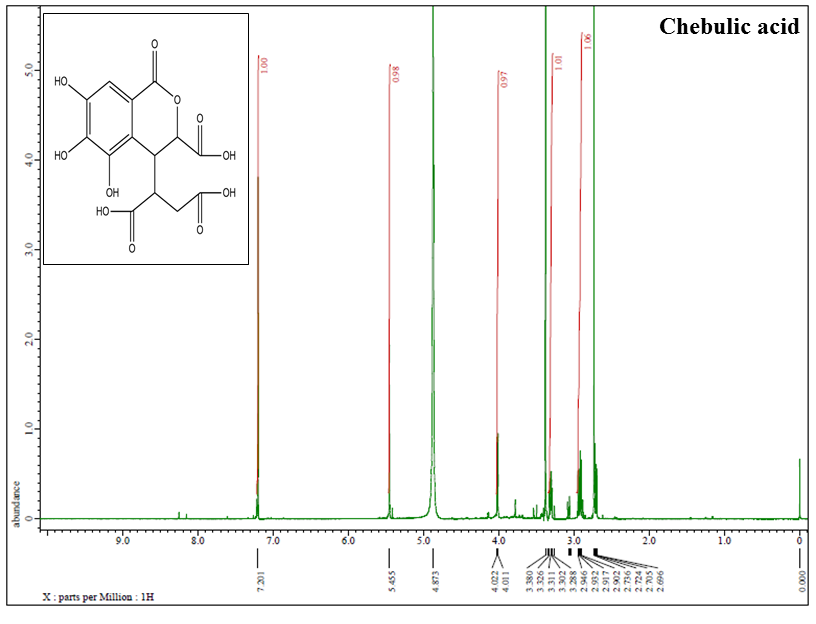
**

**Figure S4: 1H NMR analysis of compound 2**

**
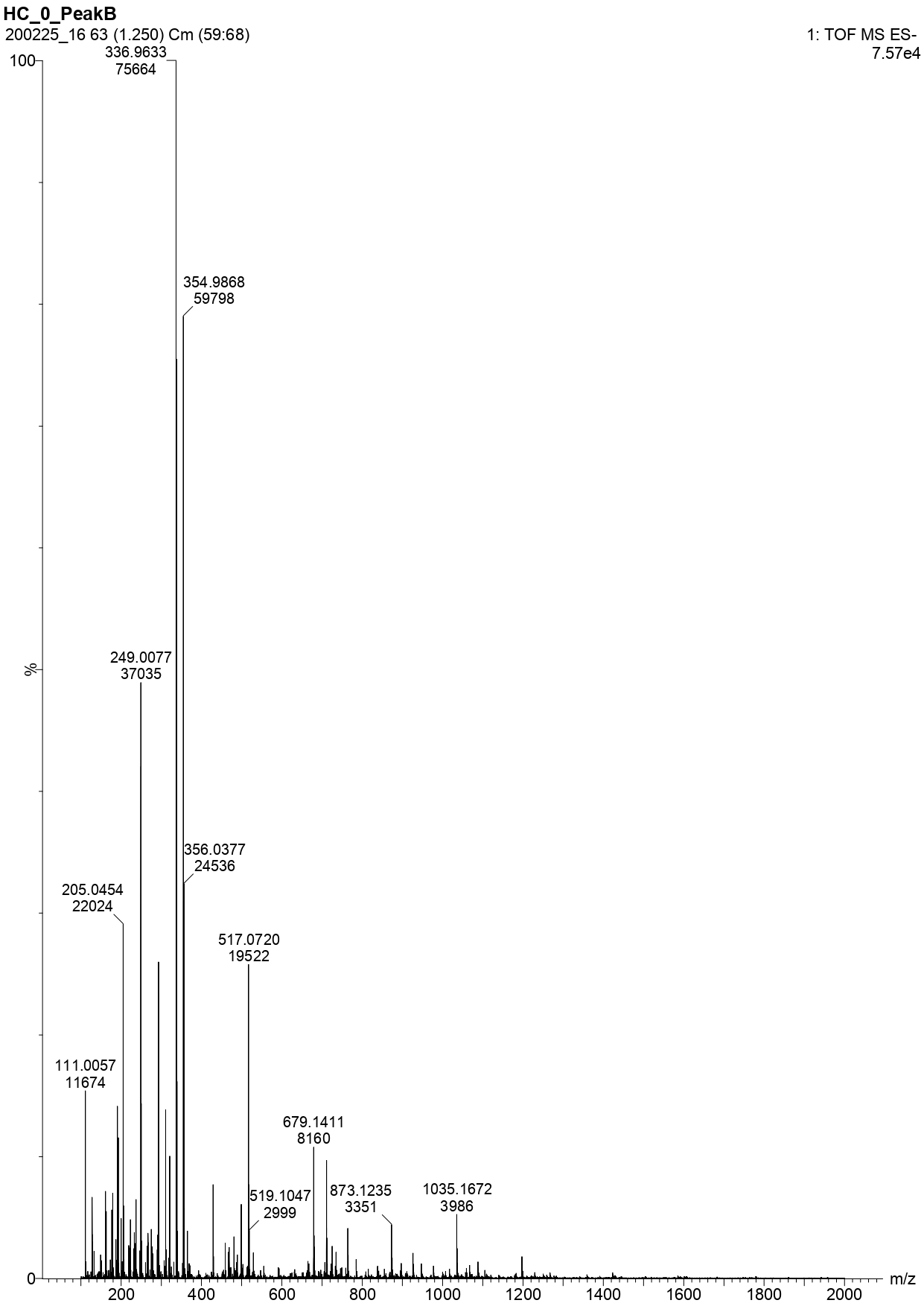
**

**Figure S5: Mass spectra analysis of compound 2**

**Compound 3
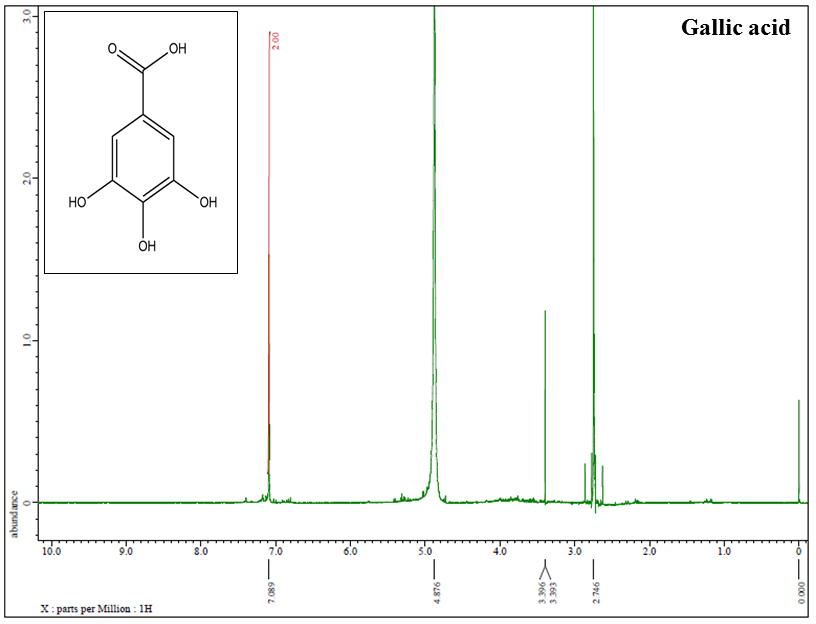
**

**Figure S6: 1H NMR analysis of compound 3**

**
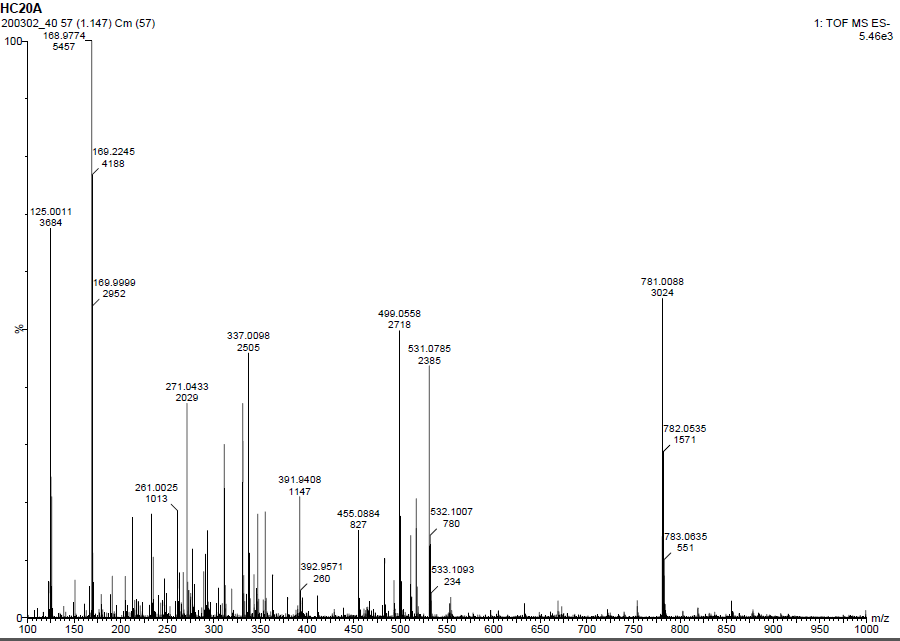
**

**Figure S7: Mass spectra analysis of compound 3**

**Compound 4**

**
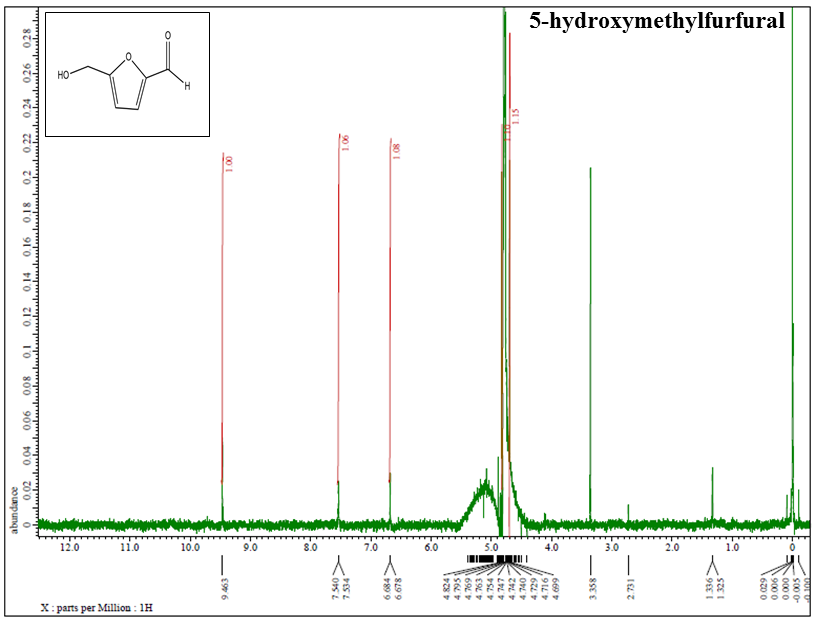
**

**Figure S8: 1H NMR analysis of compound 4**

**
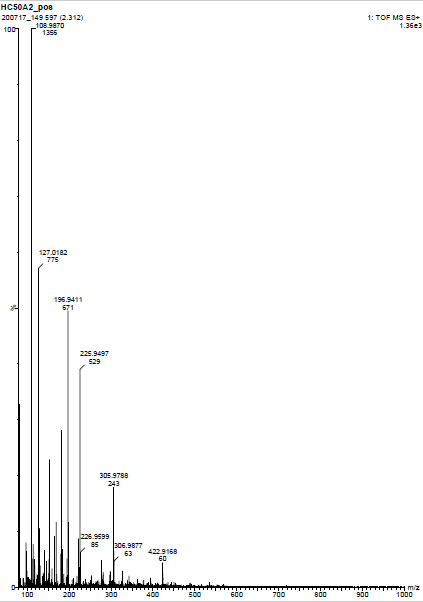
**

**Figure S9: Mass spectra analysis of compound 4**

**Compound 5**

**
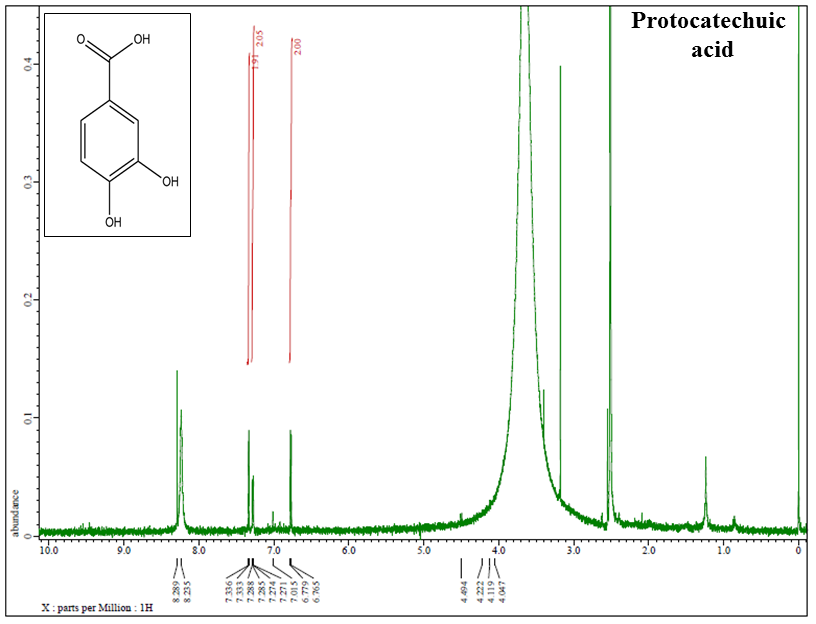
**

**Figure S10: 1H NMR analysis of compound 5**

**
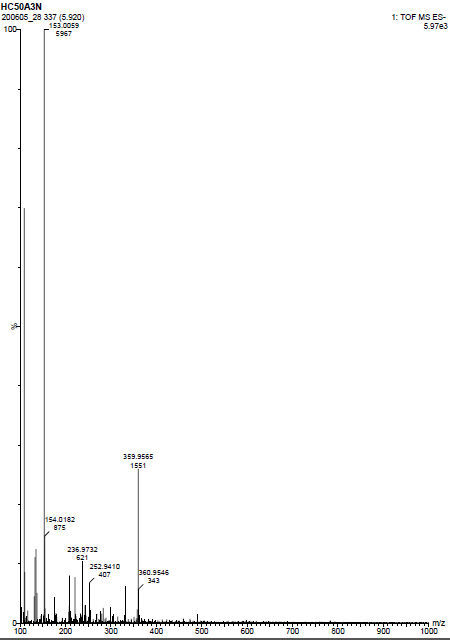
**

**Figure S11: Mass spectra analysis of compound 5**

**Compound 6**

**
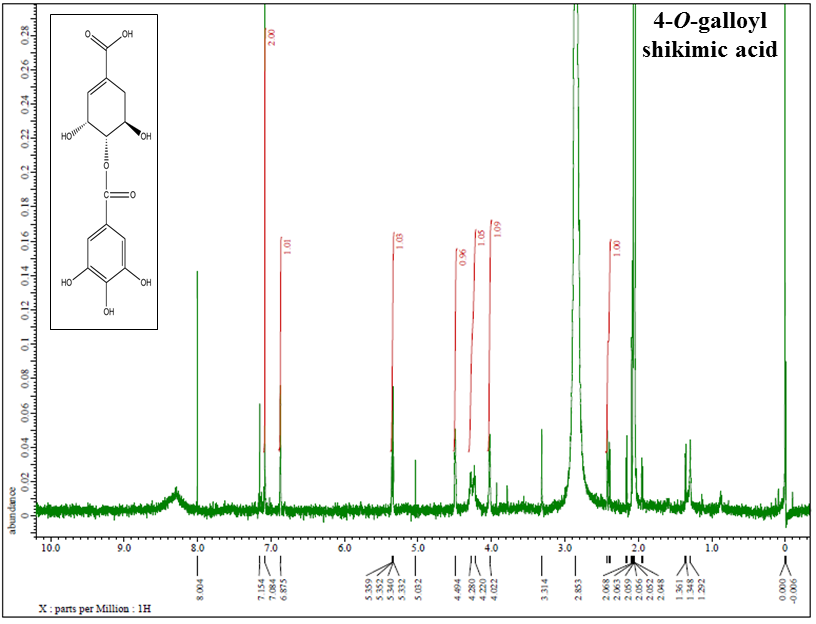
**

**Figure S12: 1H NMR analysis of compound 6 (Acetone d6)**

**
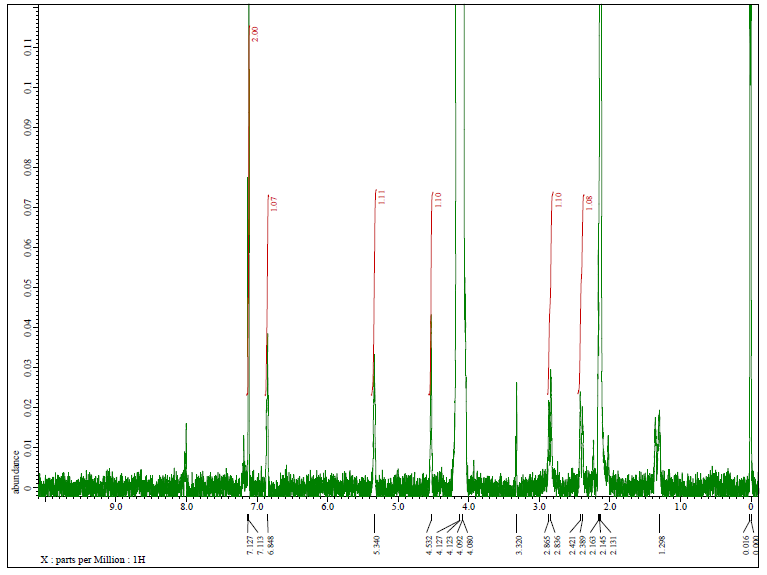
**

**Figure S13: 1H NMR analysis of compound 6 (Acetone d6 + D2O)**

**
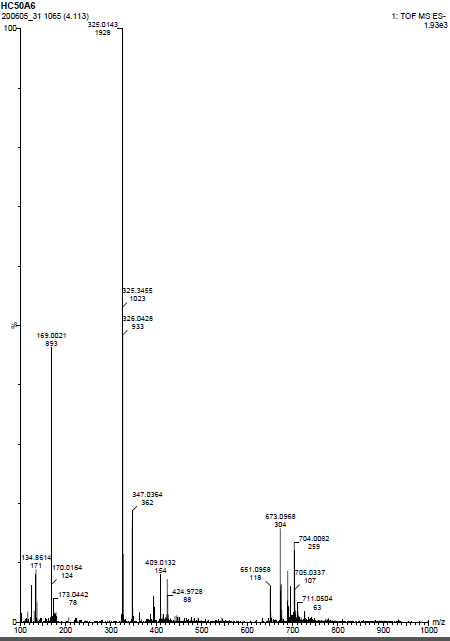
**

**Figure S14: Mass spectra analysis of compound 6**

**Compound 7**

**
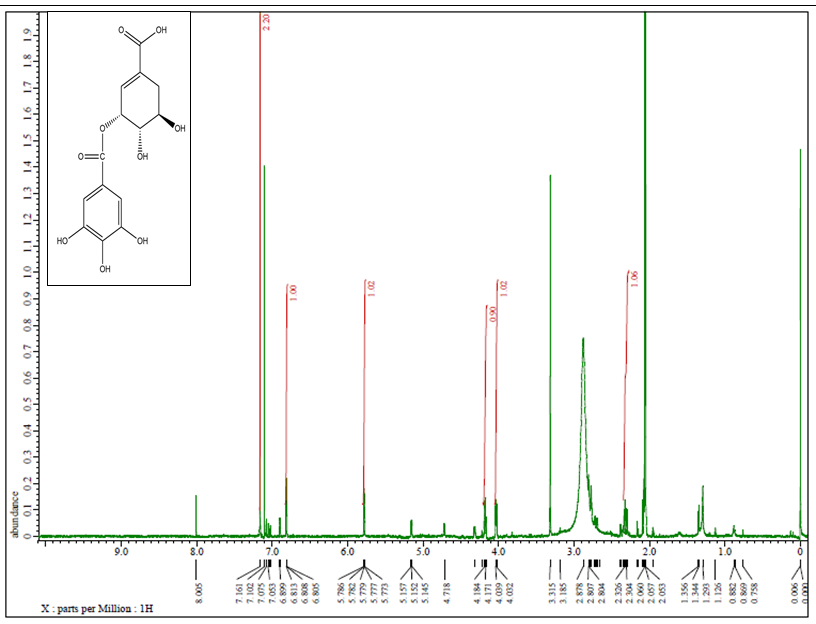
**

**Figure S15: 1H NMR analysis of compound 7**

**
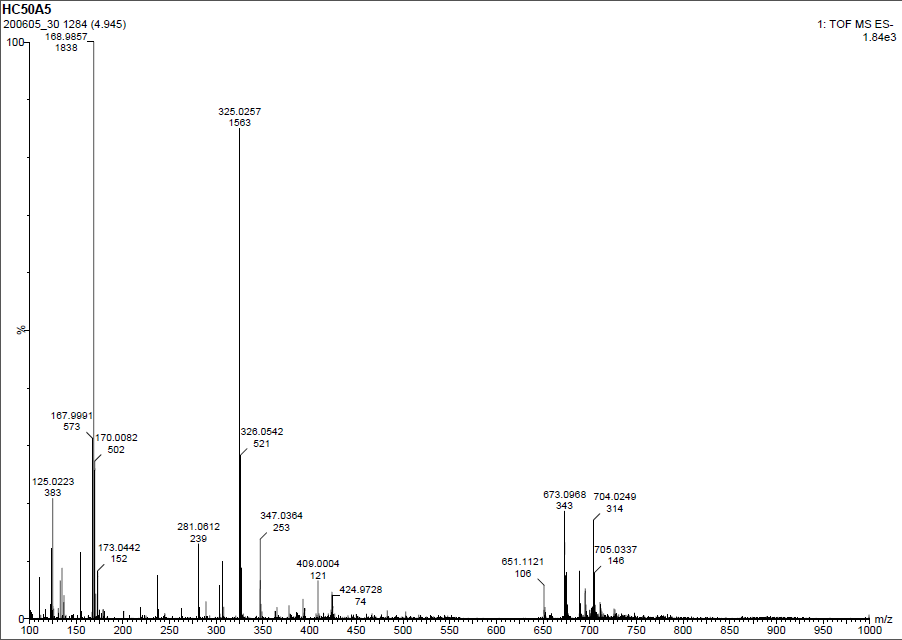
**

**Figure S16: Mass spectra analysis of compound 7**

**Compound 8**

**
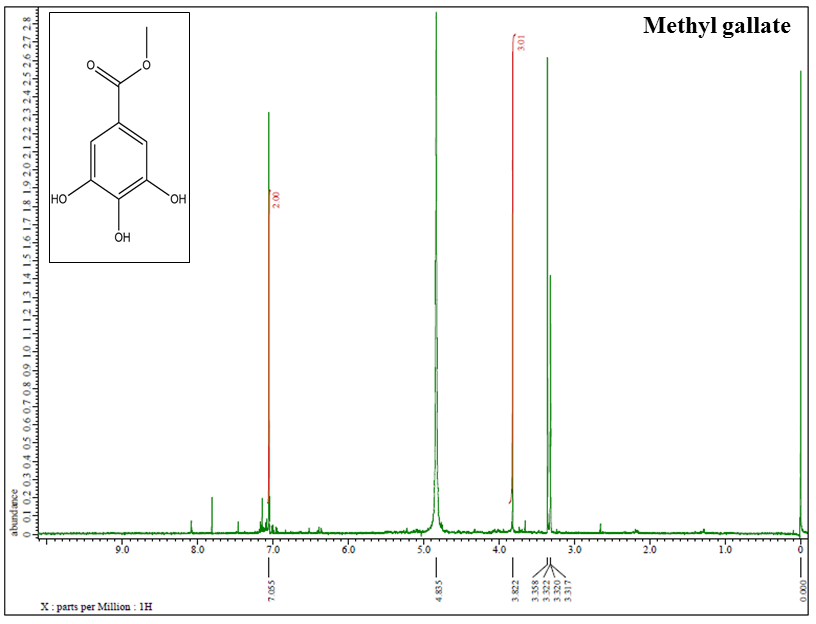
**

**Figure S17: 1H NMR analysis of compound 8**

**
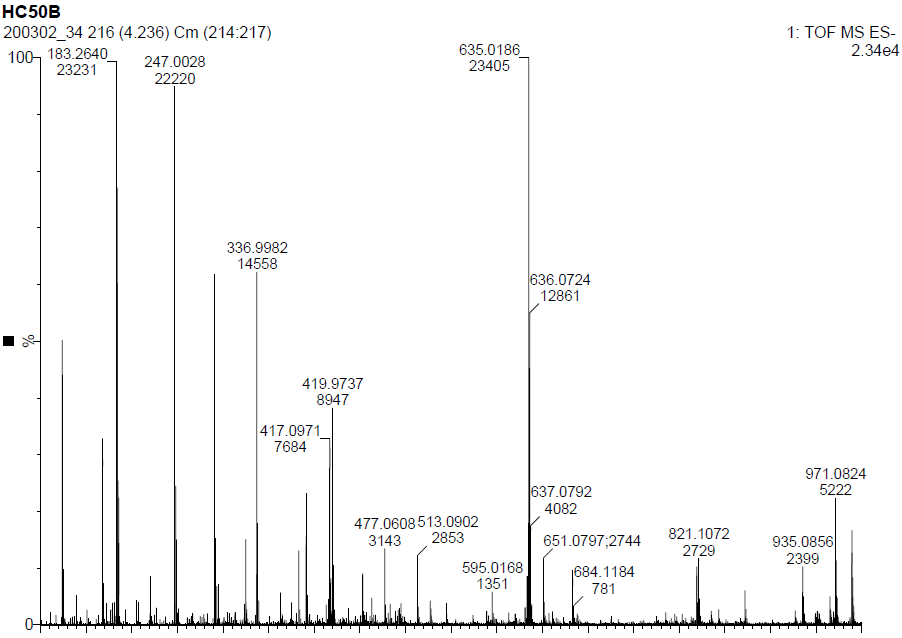
**

**Figure S18: Mass spectra analysis of compound 8**

**Compound 9**

**
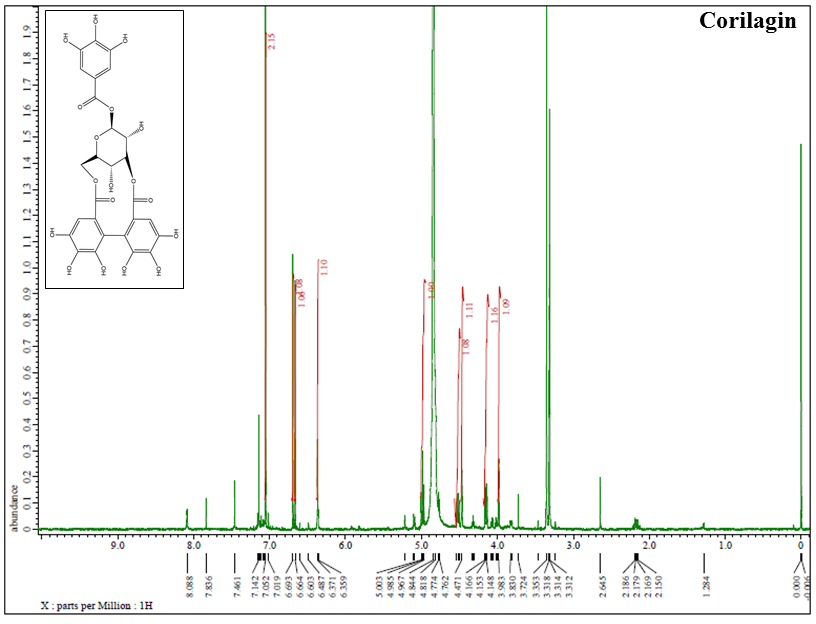
**

**Figure S19: 1H NMR analysis of compound 9**

**
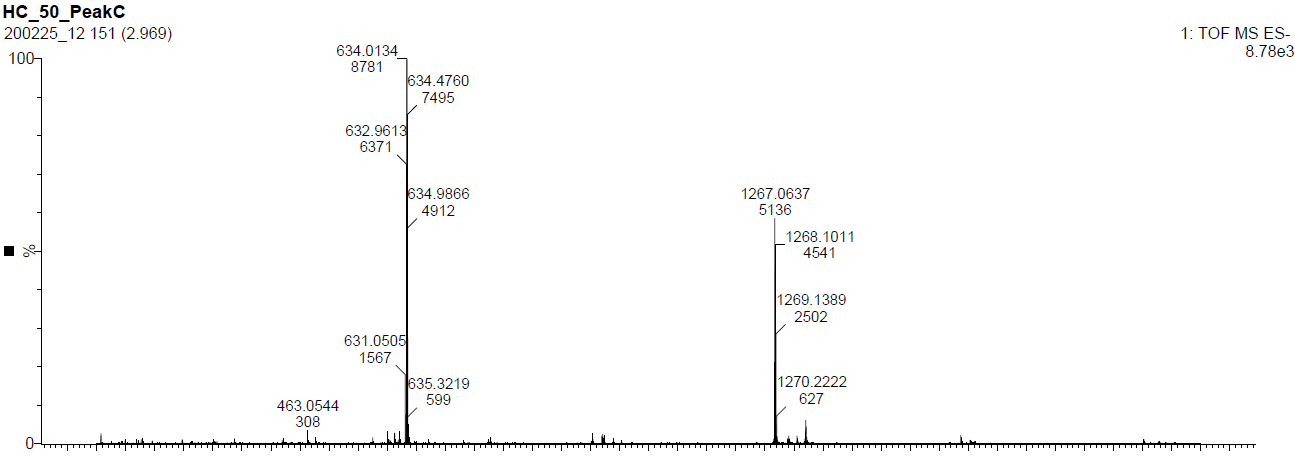
**

**Figure S20: Mass spectra analysis of compound 9**

**Compound 10**

**
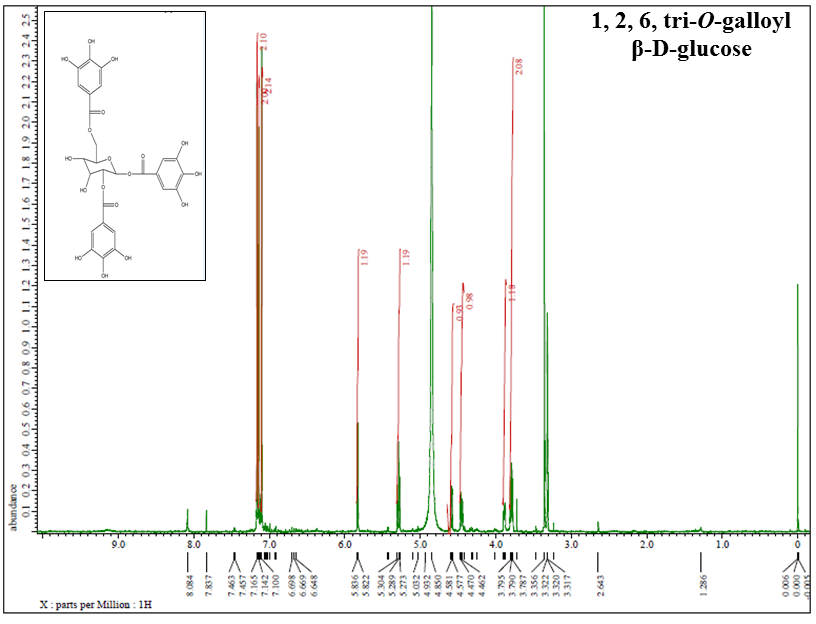
**

**Figure S21: 1H NMR analysis of compound 10**

**
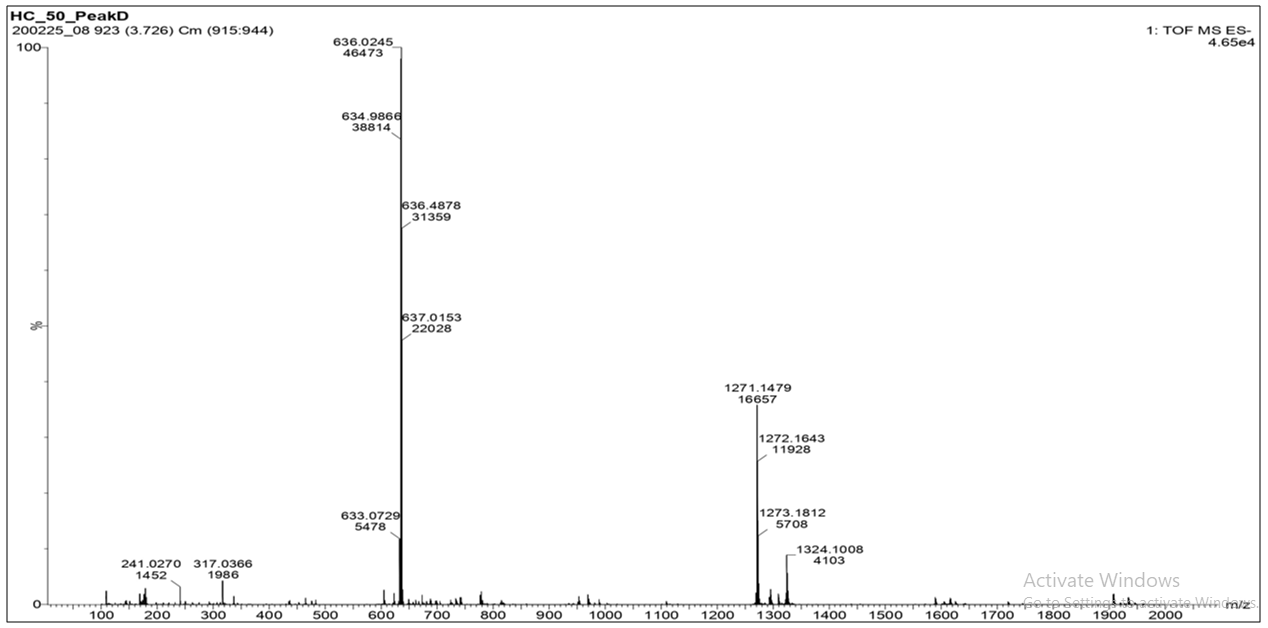
**

**Figure S22: Mass spectra analysis of compound 10**

**Compound 11**

**
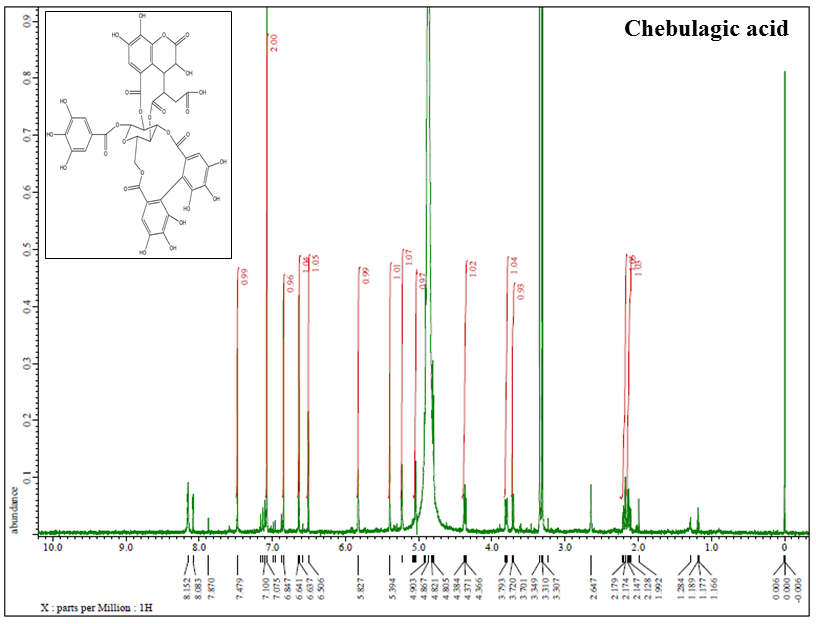
**

**Figure S23: 1H NMR analysis of compound 11**

**
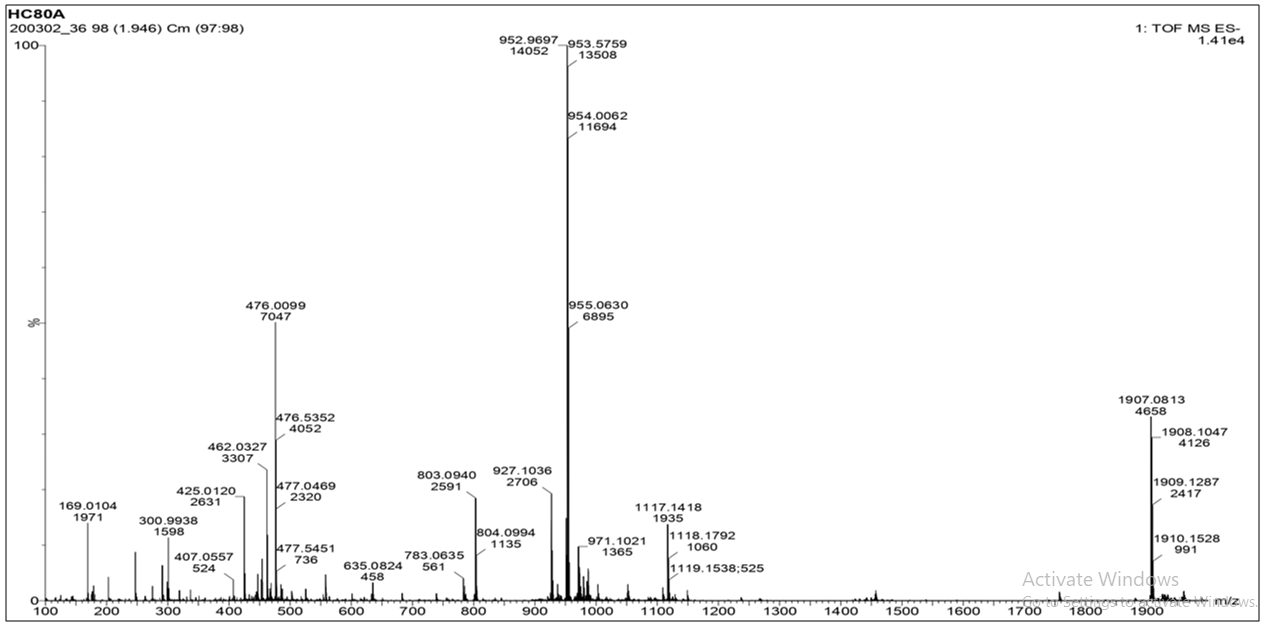
**

**Figure S24: Mass spectra analysis of compound 11**

**Compound 12**

**
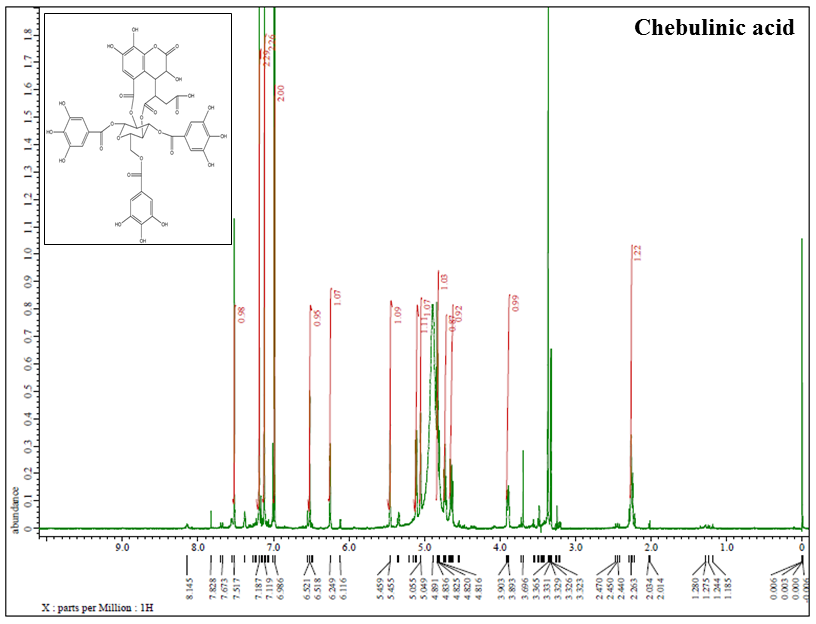
**

**Figure S25: 1H NMR analysis of compound 12**

**
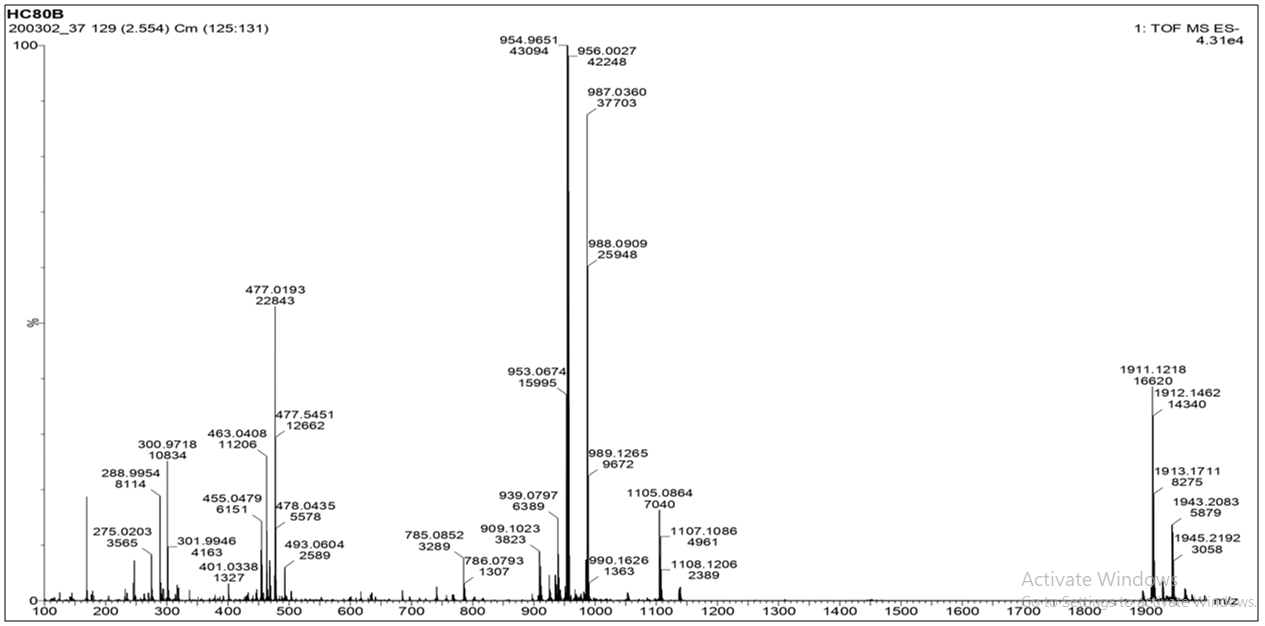
**

**Figure S26: Mass spectra analysis of compound 12**

**Compound 13**

**
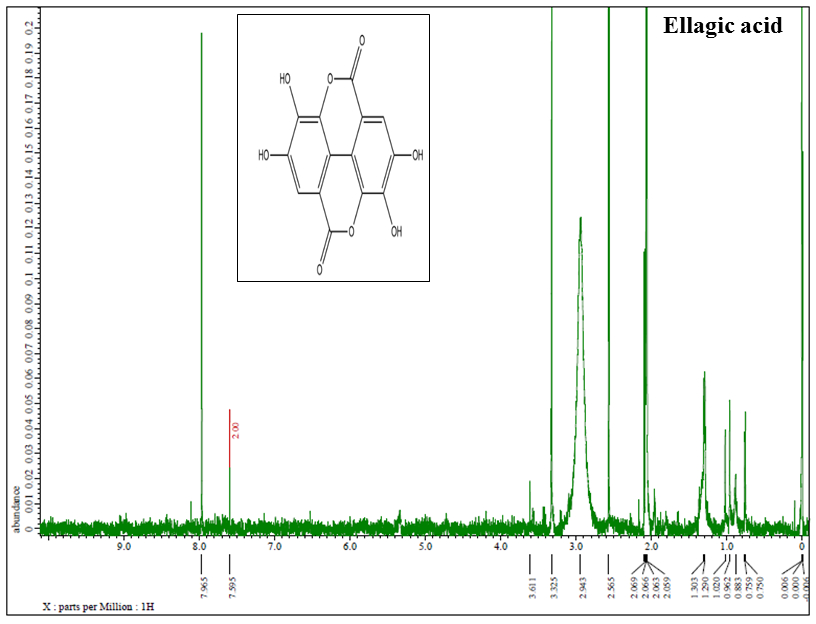
**

**Figure S27: 1H NMR analysis of compound 13**

**
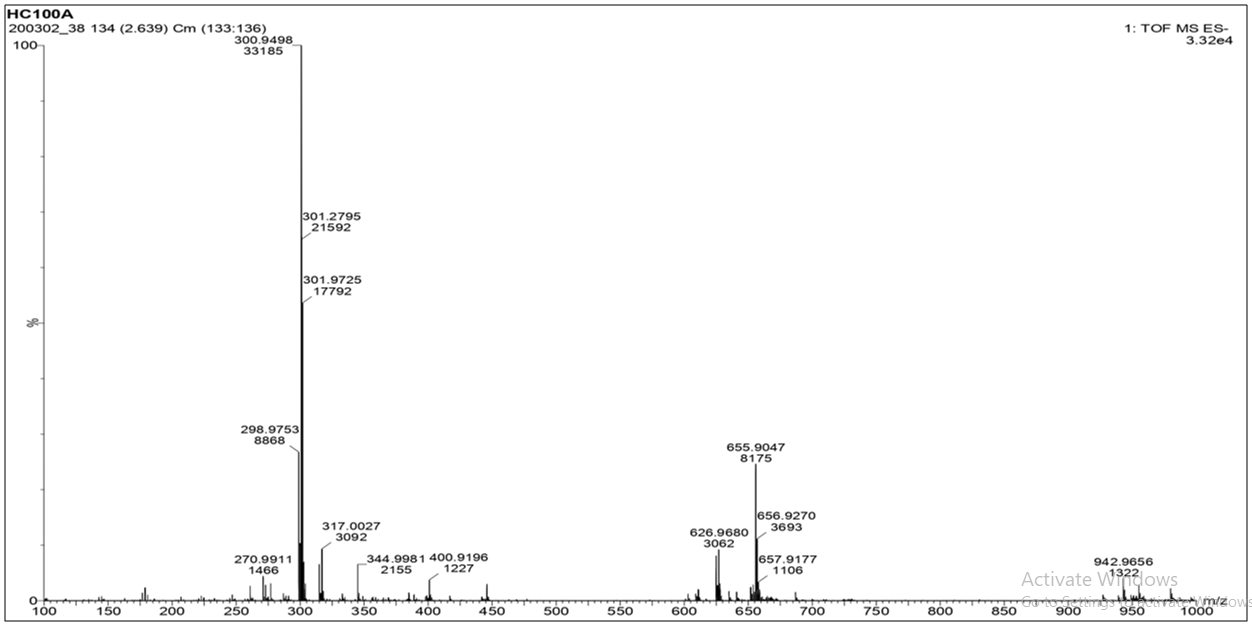
**

**Figure S28: Mass spectra analysis of compound 13**
