# Supplementary material for: Extraction, phytochemical characterization and anti-cancer mechanism of Haritaki churna: An ayurvedic formulation
Source: PLoS One. 2023 May 31;18(5):e0286274. doi: 10.1371/journal.pone.0286274 (PMC10231837; doi:10.1371/journal.pone.0286274)
Supplement: S2 Data — (DOCX) [file pone.0286274.s002.docx]

**Supplementary data-2_ Purity of compounds isolated from HCAE and quantification of GA, MG and EA in HCAE using standard curves**

Figure S1: HPLC chromatogram of isolated compound 1

Figure S2: HPLC chromatogram of isolated compound 2

Figure S3: HPLC chromatogram of isolated compound 3

Figure S4: HPLC chromatogram of isolated compound 4

Figure S5: HPLC chromatogram of isolated compound 5

Figure S6: HPLC chromatogram of isolated compound 6

Figure S7: HPLC chromatogram of isolated compound 7

Figure S8: HPLC chromatogram of isolated compound 8

Figure S9: HPLC chromatogram of isolated compound 9

Figure S10: HPLC chromatogram of isolated compound 10

Figure S11: HPLC chromatogram of isolated compound 11

Figure S12: HPLC chromatogram of isolated compound 12

Figure S13: HPLC chromatogram of isolated compound 13


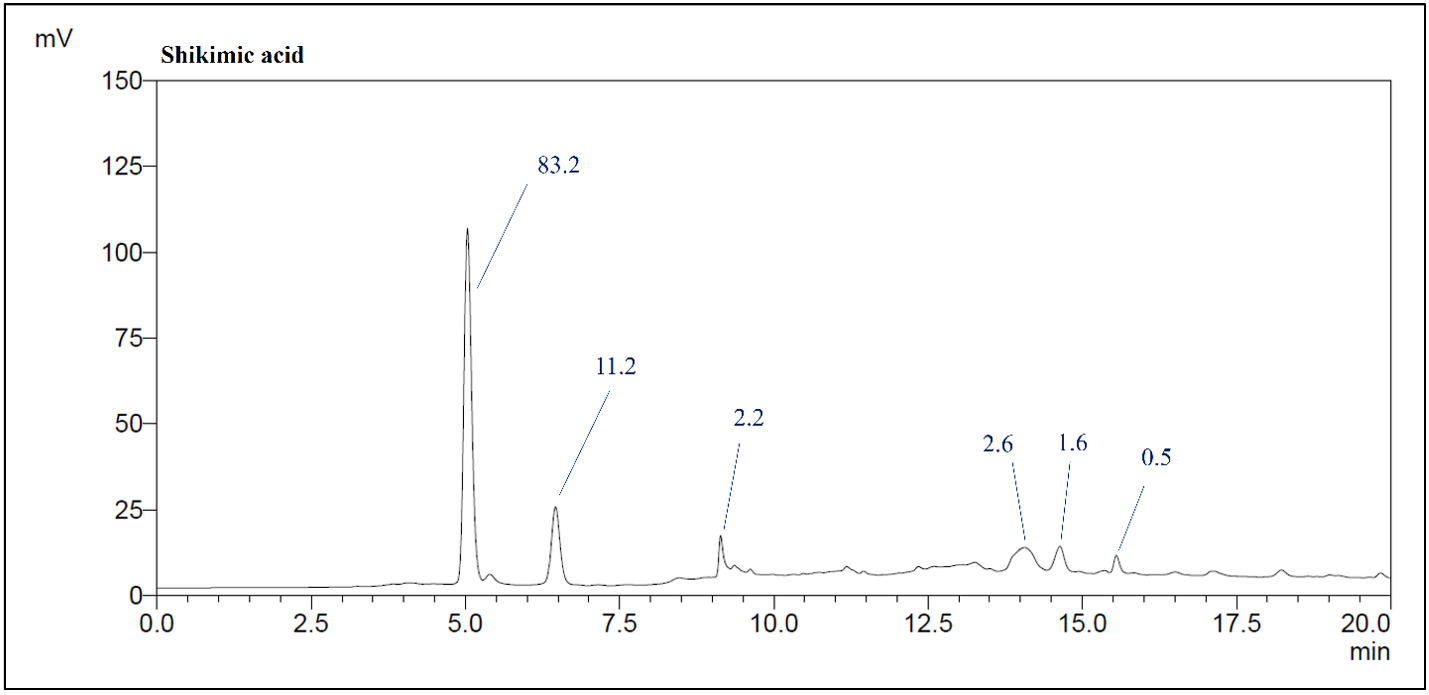


**Figure S1: HPLC chromatogram of isolated compound 1**

**
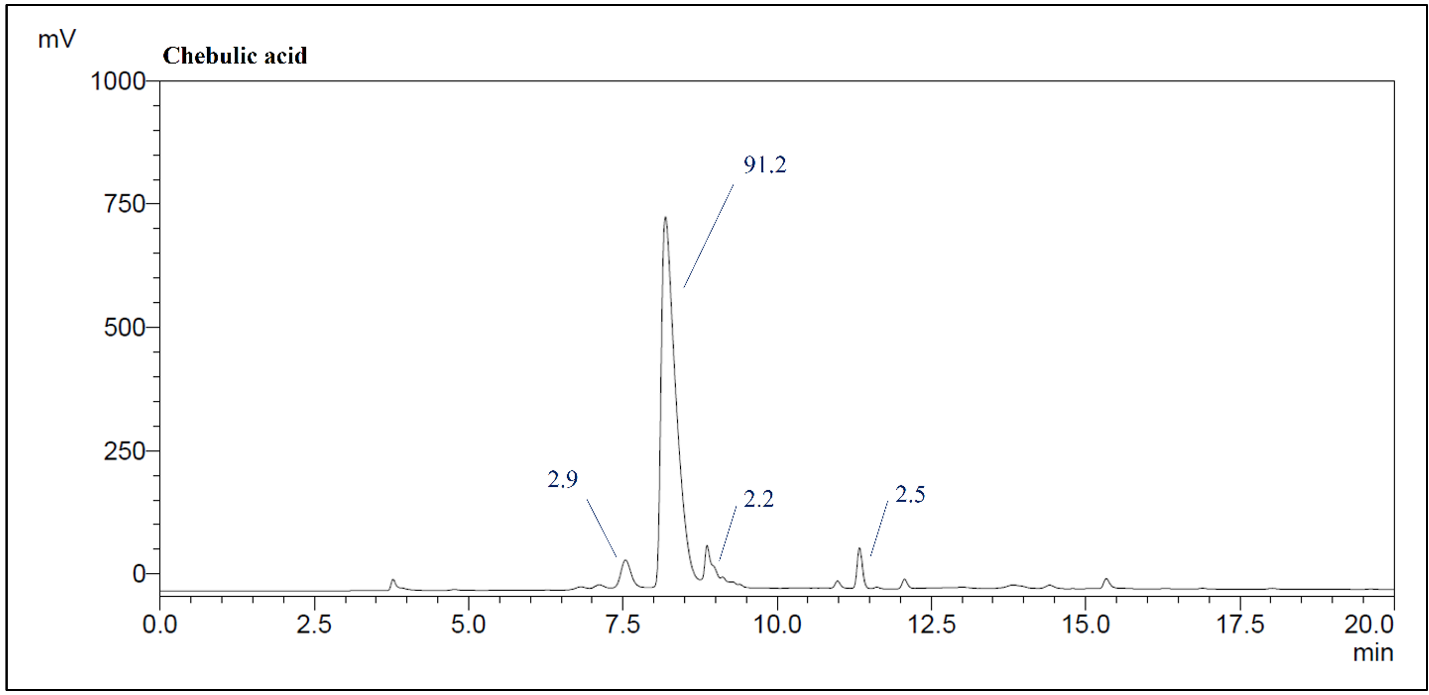
**

**Figure S2: HPLC chromatogram of isolated compound 2**


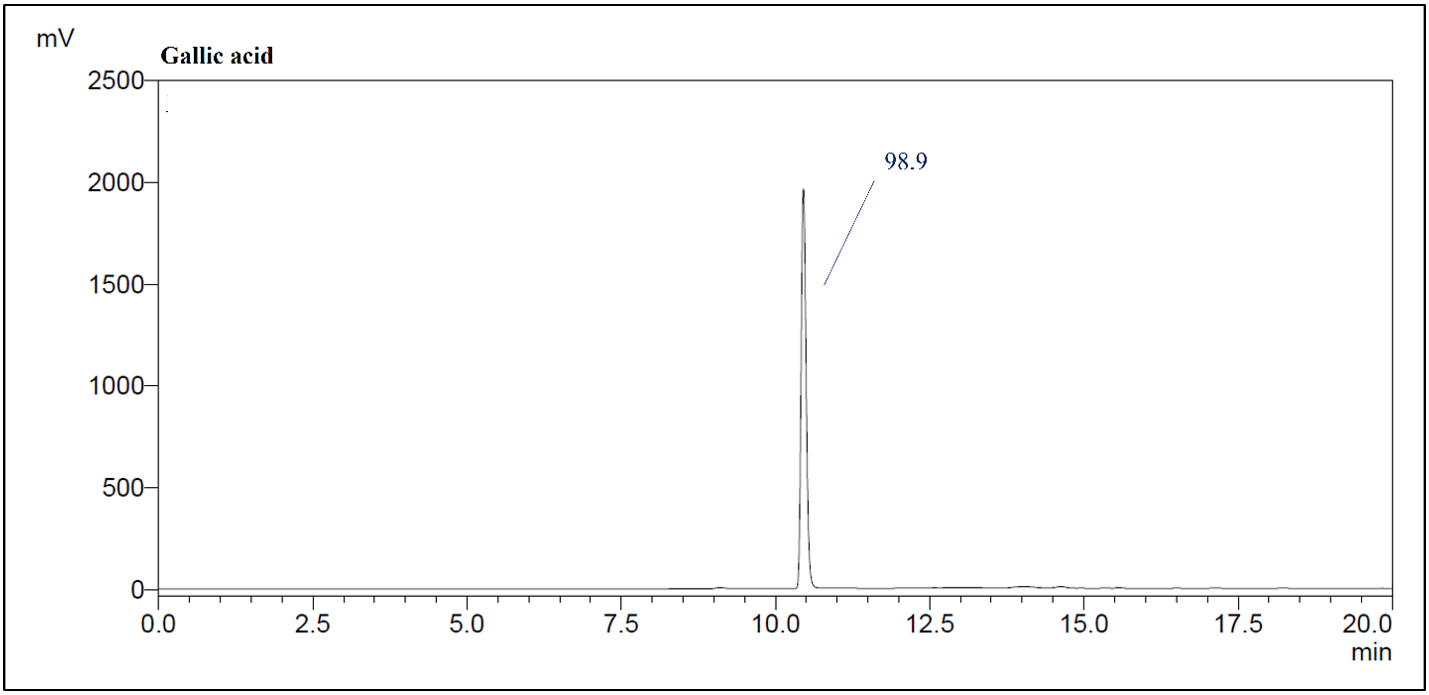


**Figure S3: HPLC chromatogram of isolated compound 3**


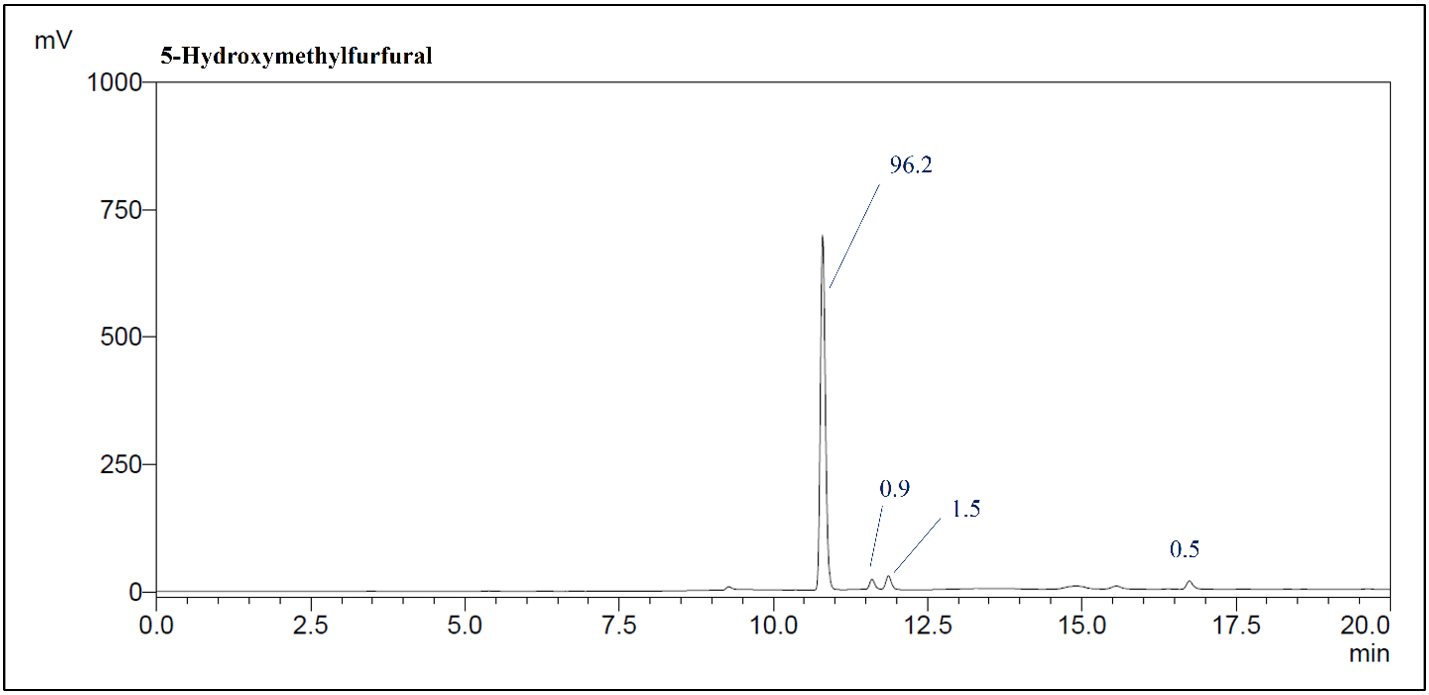


**Figure S4: HPLC chromatogram of isolated compound 4**


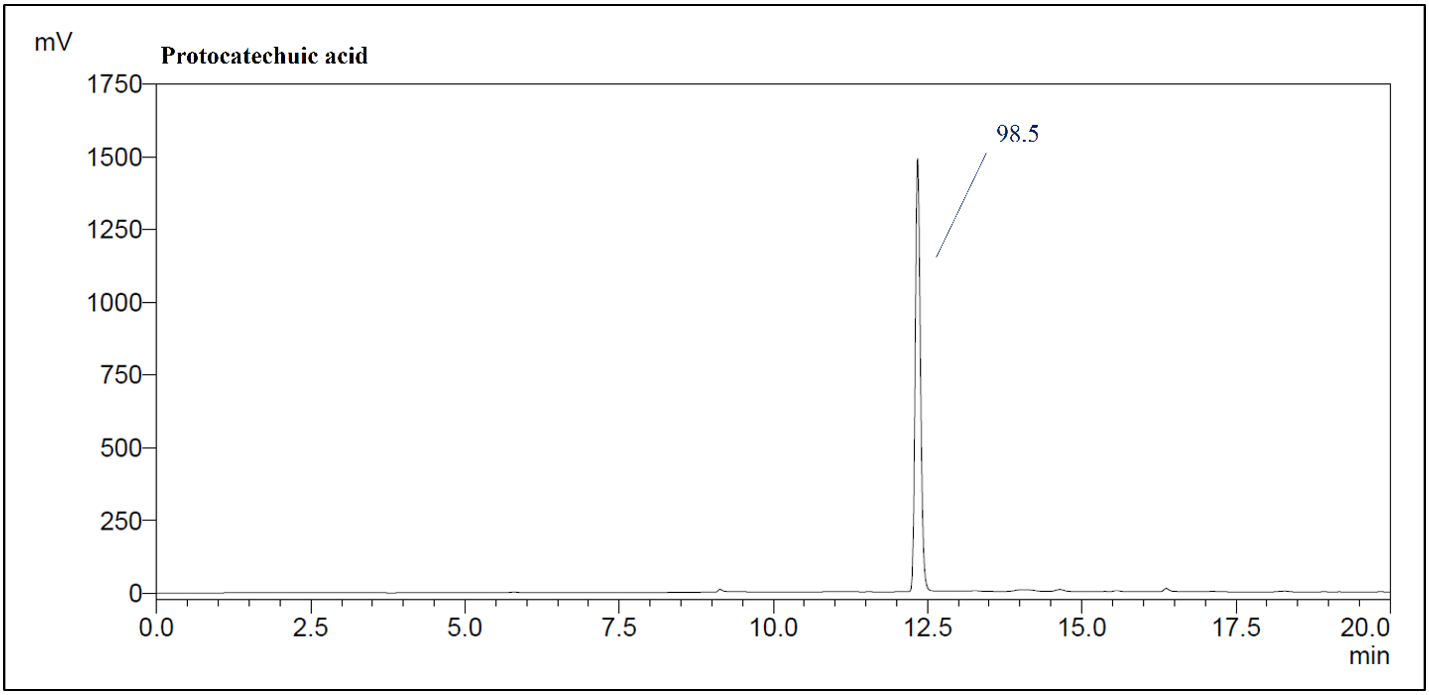


**Figure S5: HPLC chromatogram of isolated compound 5**


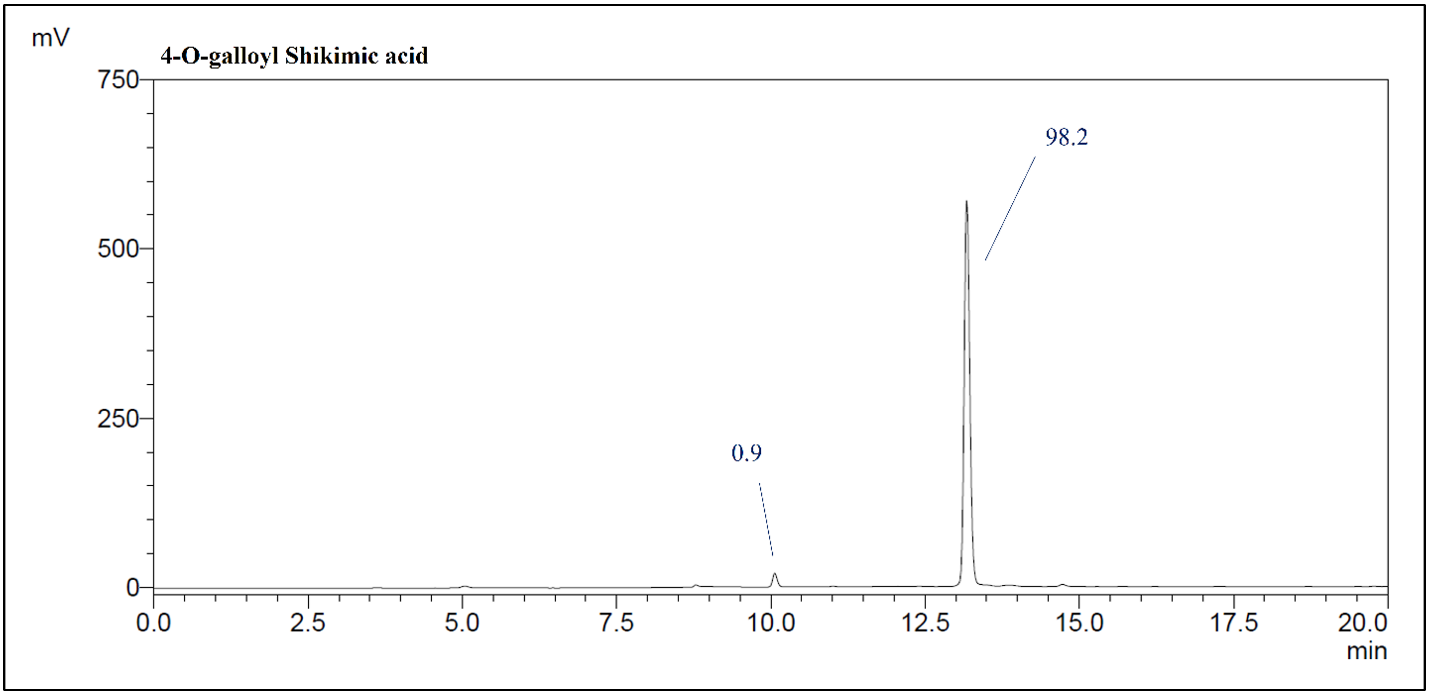


**Figure S6: HPLC chromatogram of isolated compound 6**


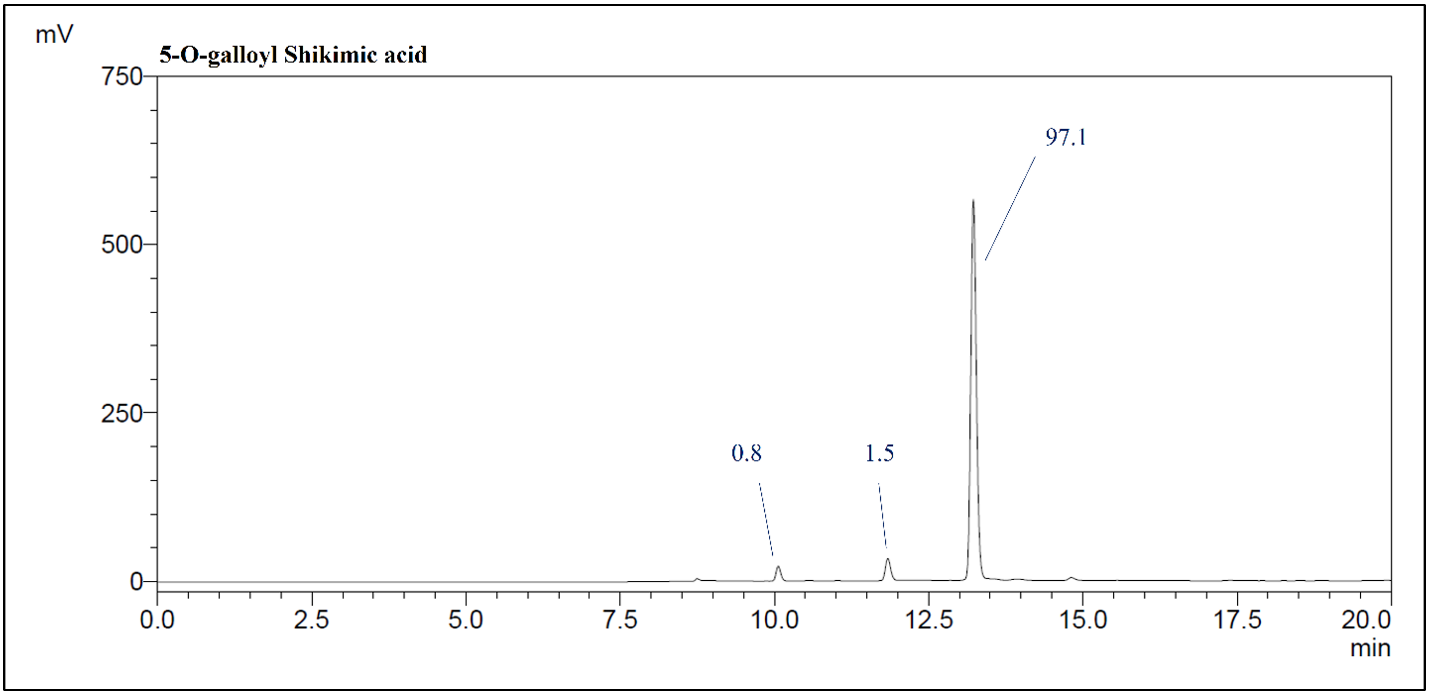


**Figure S7: HPLC chromatogram of isolated compound 7**


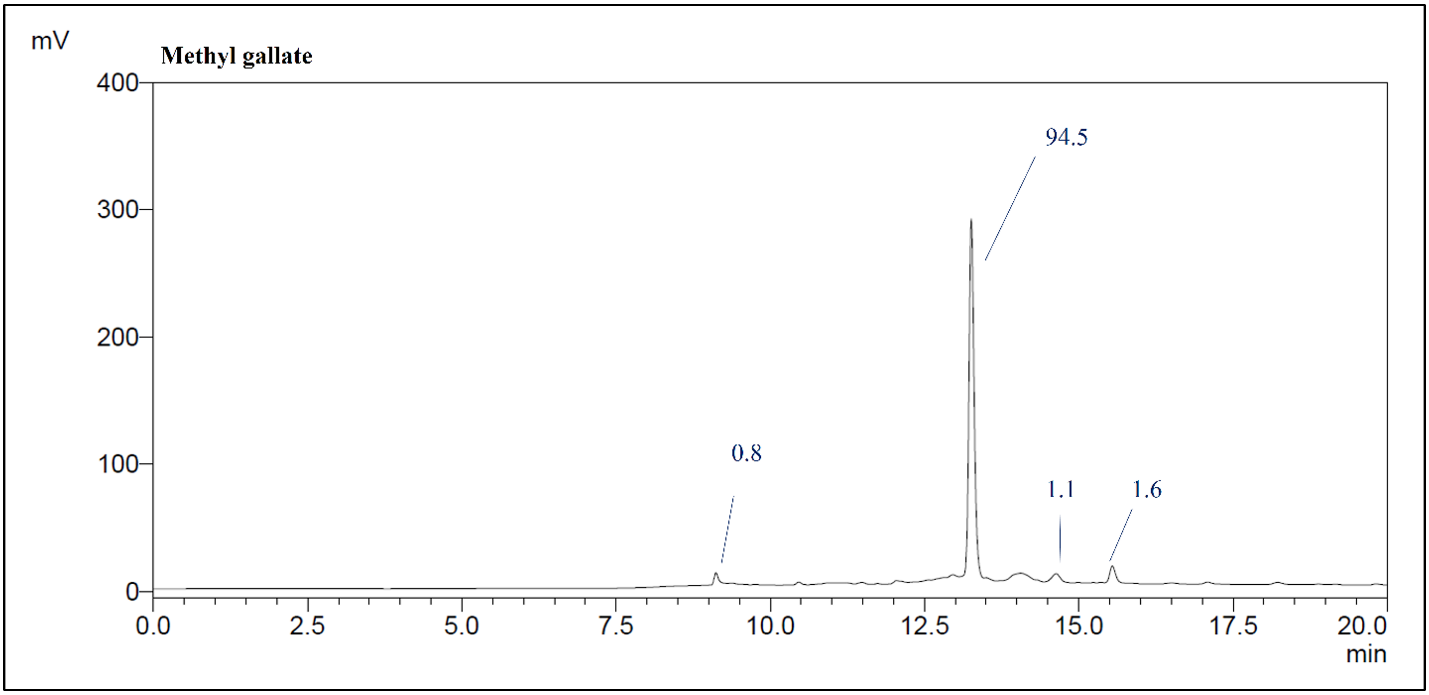


**Figure S8: HPLC chromatogram of isolated compound 8**


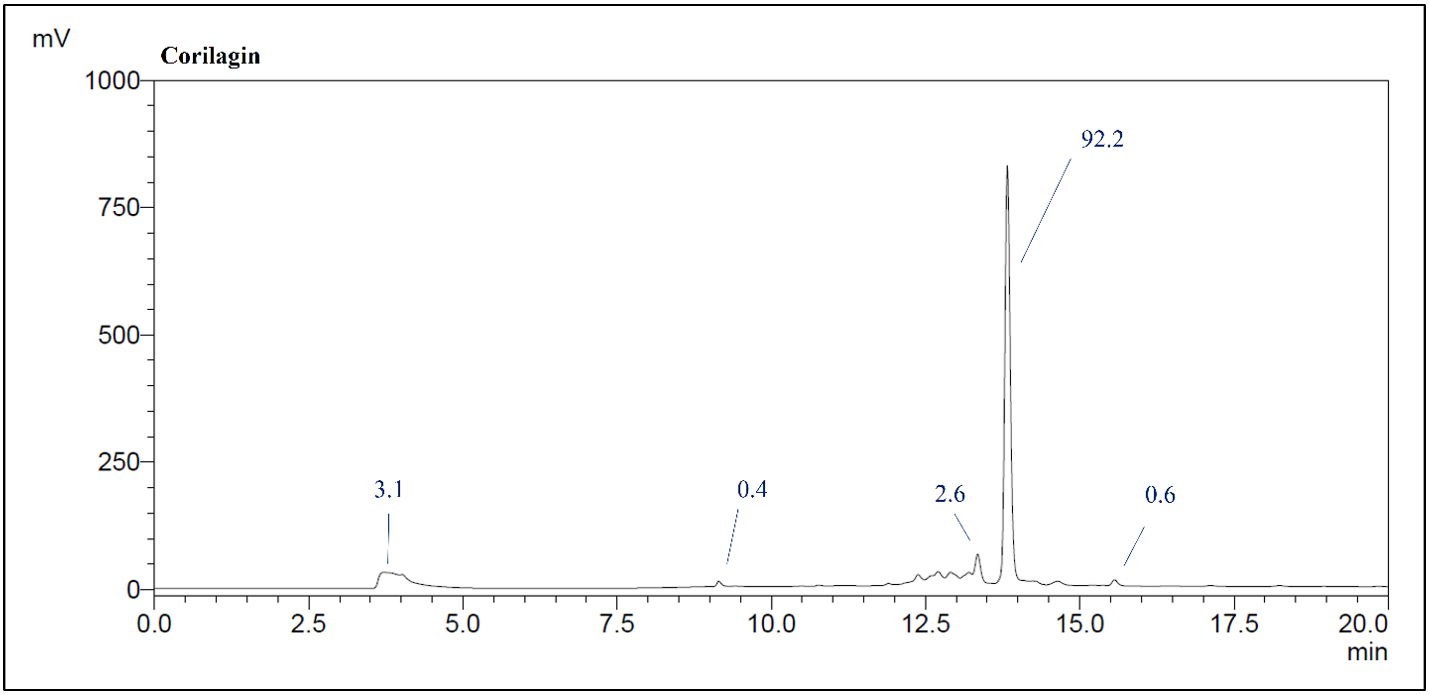


**Figure S9: HPLC chromatogram of isolated compound 9**


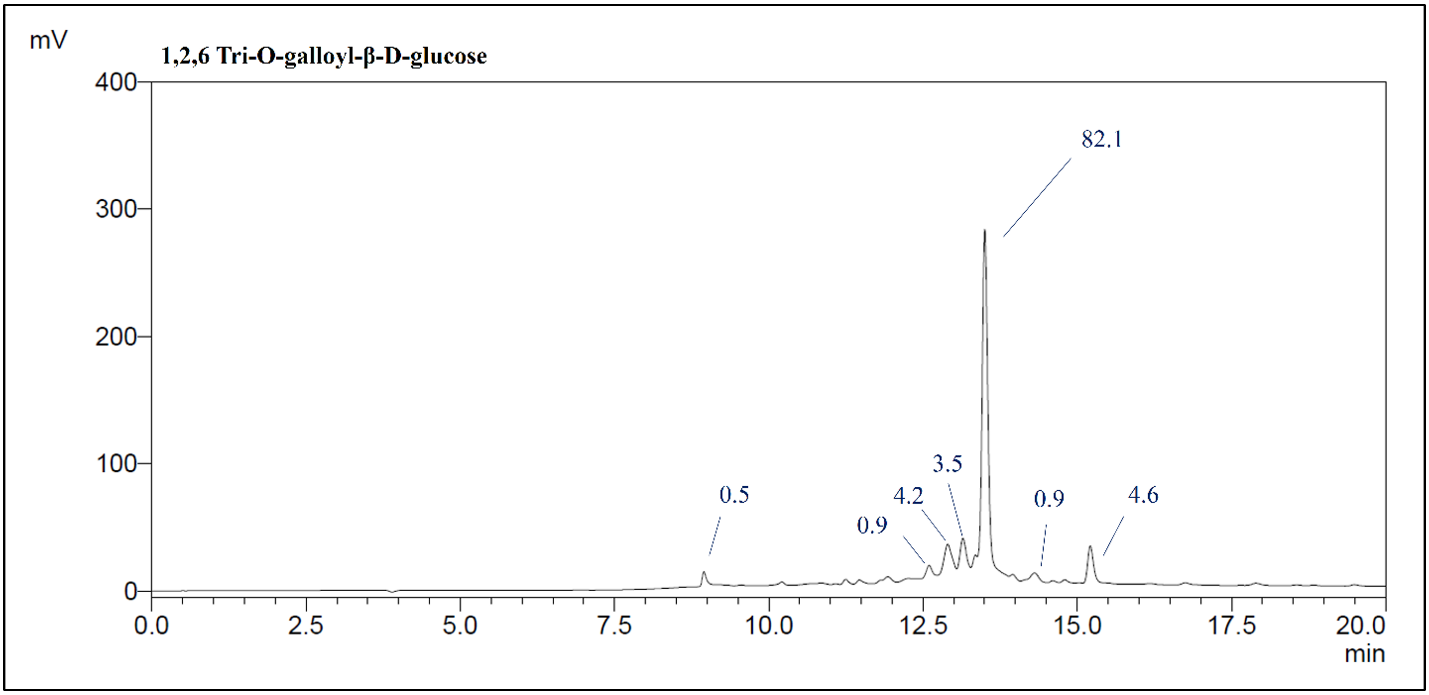


**Figure S10: HPLC chromatogram of isolated compound 10**


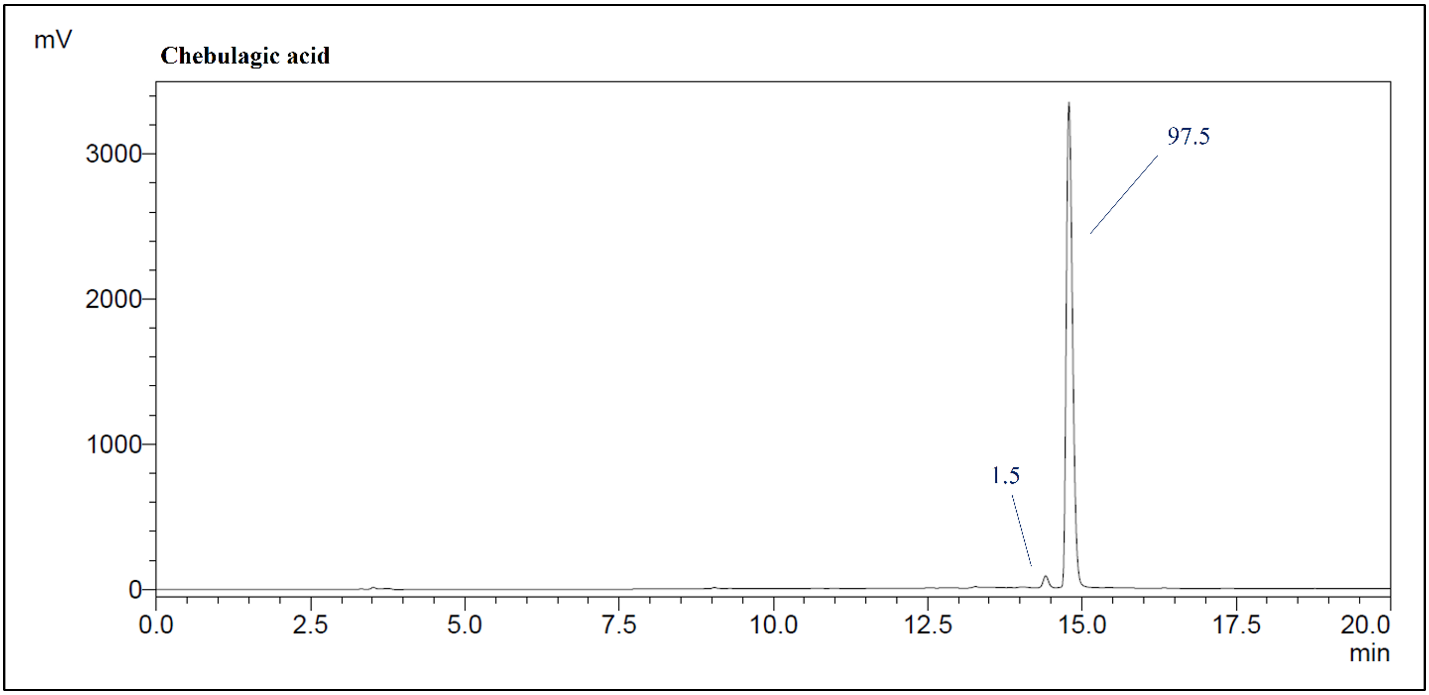


**Figure S11: HPLC chromatogram of isolated compound 11**


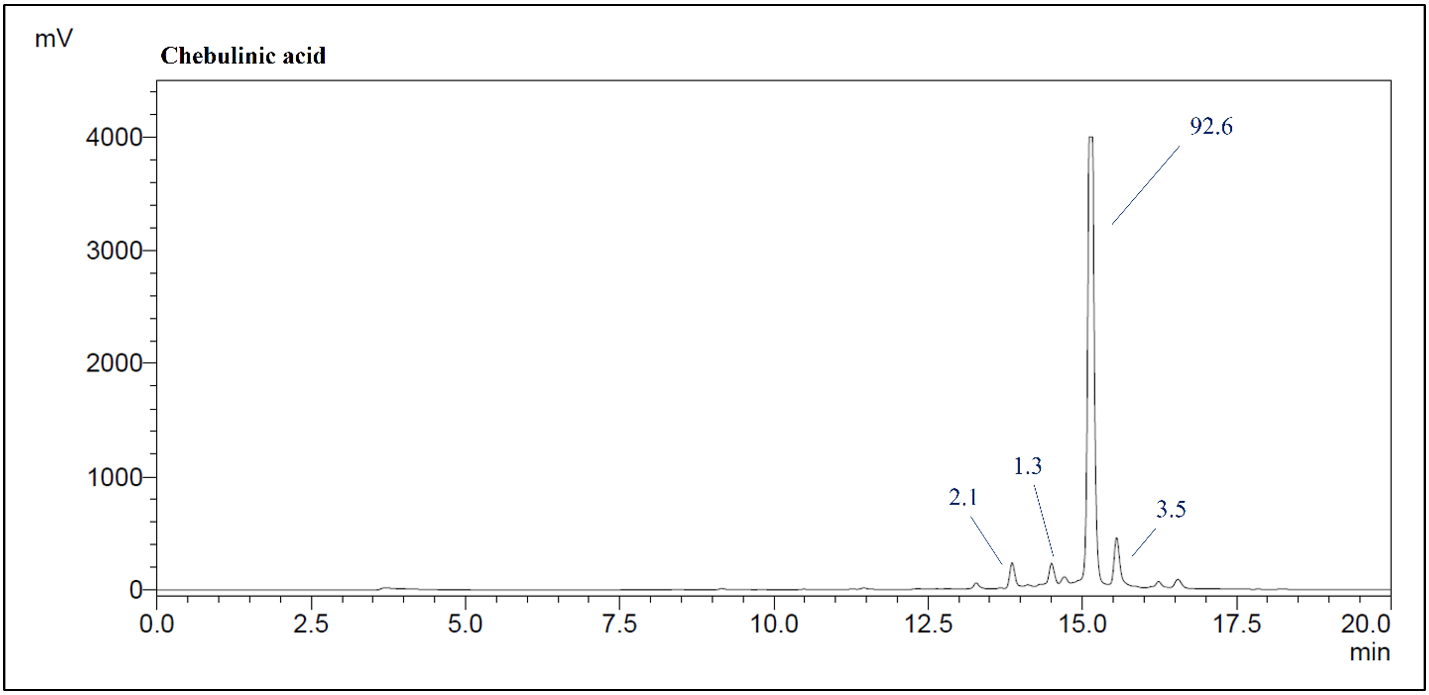


**Figure S12: HPLC chromatogram of isolated compound 12**


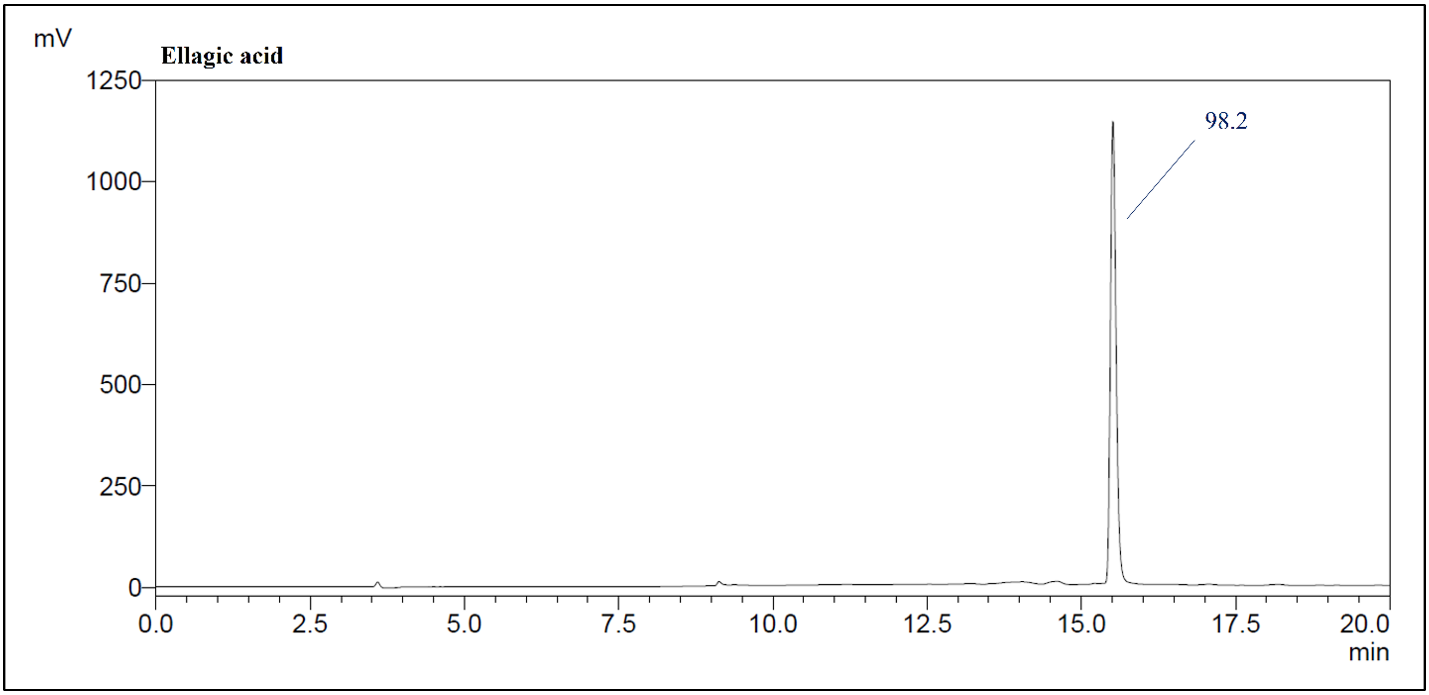


**Figure S13: HPLC chromatogram of isolated compound 13**





**Figure S14: HPLC chromatogram Gallic acid standard at various concentrations**





**Figure S15: HPLC chromatogram Methyl gallate standard at various concentrations**





**Figure S16: HPLC chromatogram Ellagic acid standard at various concentrations**

**Quantification of Gallic acid, methyl gallate and ellagic acid from HCAE using standard curved**





**Figure S17: Standard curves for gallic acid, methyl gallate and ellagic acid used for their quantification in HCAE crude extract**

Linear curve for gallic acid standard: **Y= 2.99 + 2.32 X**

Linear curve for gallic acid standard: **Y= -10.44 + 1.25 X**

Linear curve for gallic acid standard: **Y= -10.11 + 4.7 X**
