# Supplementary material for: Extraction, phytochemical characterization and anti-cancer mechanism of Haritaki churna: An ayurvedic formulation
Source: PLoS One. 2023 May 31;18(5):e0286274. doi: 10.1371/journal.pone.0286274 (PMC10231837; doi:10.1371/journal.pone.0286274)
Supplement: S3 Data — (DOCX) [file pone.0286274.s003.docx]

**Supplementary data-3_ Cell viability assays for fractions and compounds isolated from HCAE against colorectal cancer (DLD1, HCT-116 and HT-29) cell lines**

**Figure S1**: Cell Viability graphs for HCAE fractions and compounds against HCT-116 cell line after 48 hours of treatment

**Figure S2**: Cell Viability graphs for HCAE fractions and compounds against DLD1 cell line after 48 hours of treatment

**Figure S3**: Cell Viability graphs for HCAE fractions and compounds against HT-29 cell line after 48 hours of treatment


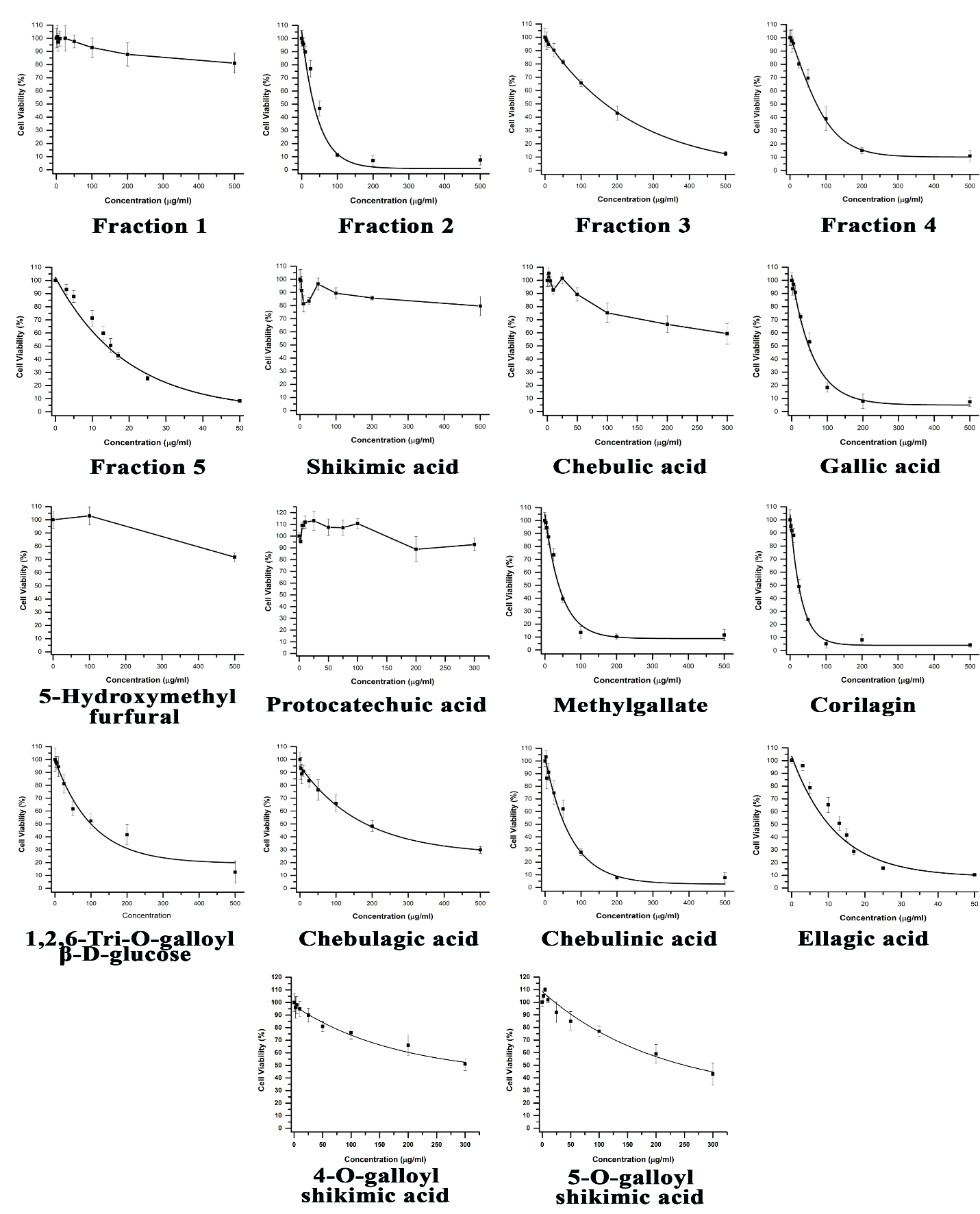


**Figure S1**: Cell Viability graphs for HCAE fractions and compounds against HCT-116 cell line after 48 hours of treatment

**
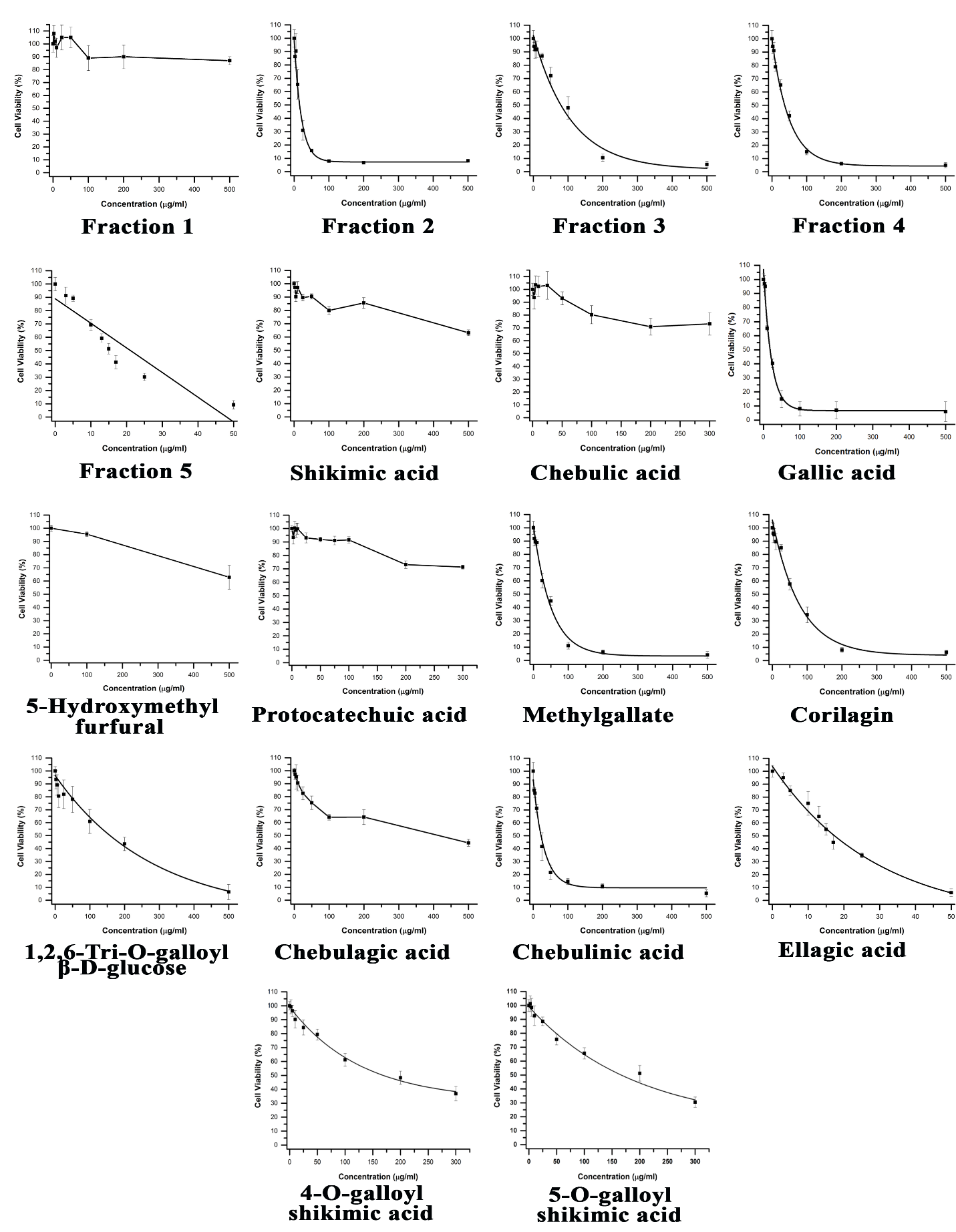
**

**Figure S2**: Cell Viability graphs for HCAE fractions and compounds against DLD1 cell line after 48 hours of treatment

**
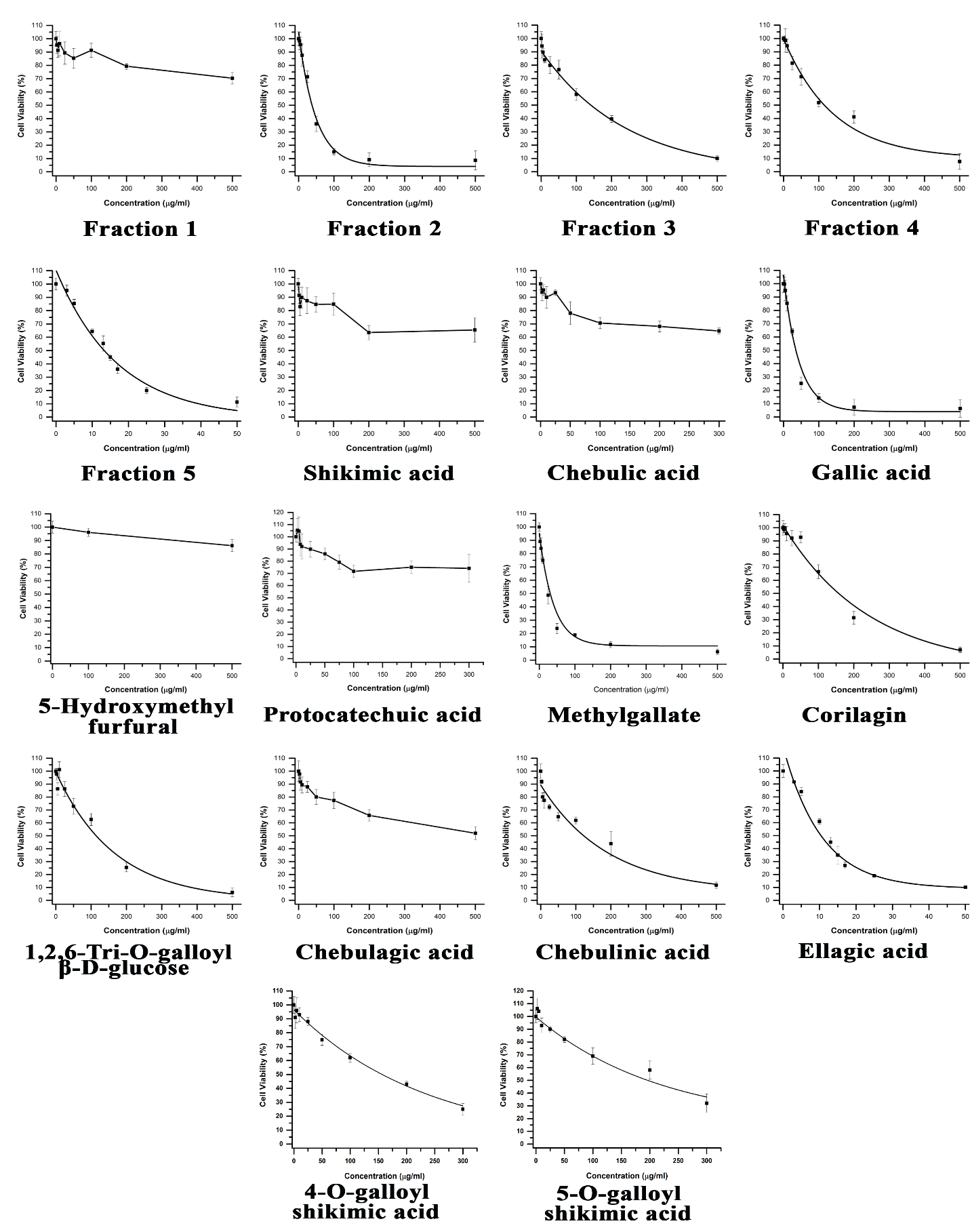
**

**Figure S3**: Cell Viability graphs for HCAE fractions and compounds against HT-29 cell line after 48 hours of treatment
