# Supplementary material for: Extraction, phytochemical characterization and anti-cancer mechanism of Haritaki churna: An ayurvedic formulation
Source: PLoS One. 2023 May 31;18(5):e0286274. doi: 10.1371/journal.pone.0286274 (PMC10231837; doi:10.1371/journal.pone.0286274)

Supplementary data-4_Raw images of western blots

Bcl2 protein after 24 & 48-hour treatment with ellagic acid

Developed blot (24 Hour)

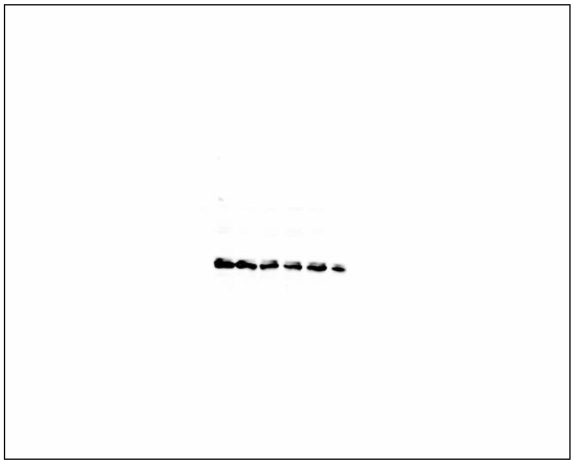

Colorimetric blot (24 Hour)

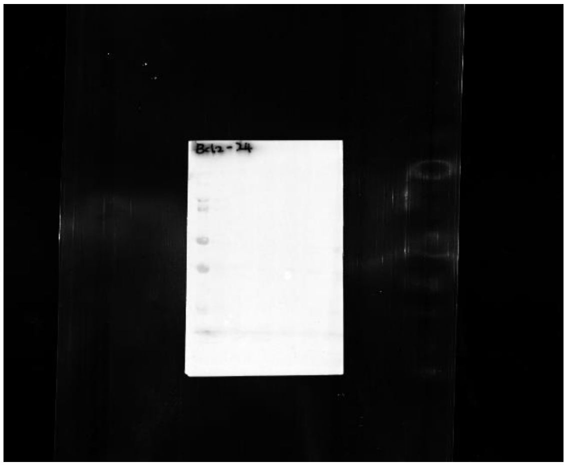

Developed blot (48 Hour)

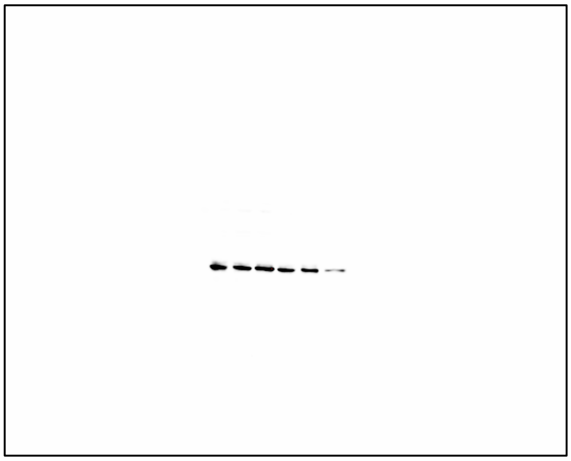

Colorimetric blot (48 Hour)

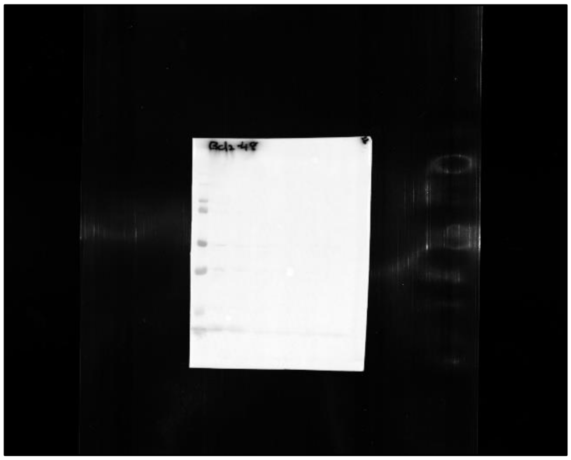

Caspase 3 and cleaved caspase 3 protein after 24 & 48-hour treatment with ellagic acid

Developed blot (24 Hour)

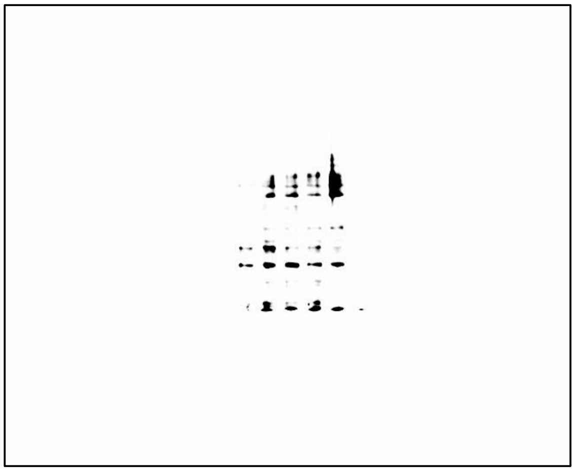

Colorimetric blot (24 Hour)

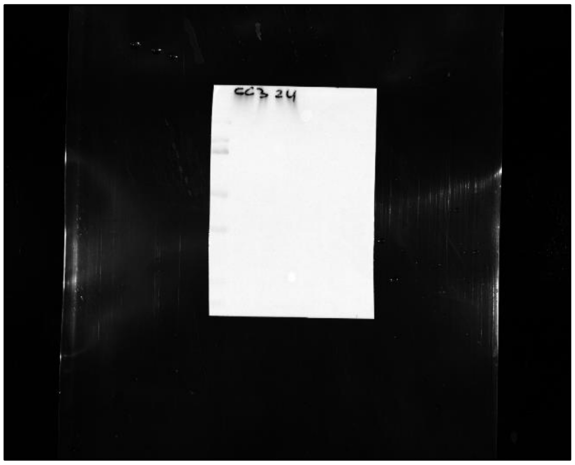

Developed blot (48 Hour)

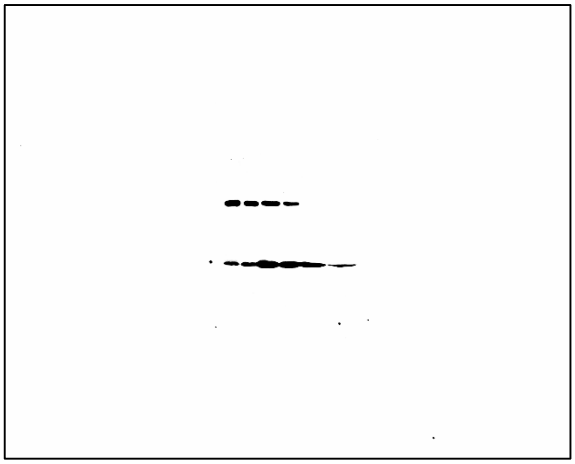

Colorimetric blot (48 Hour)

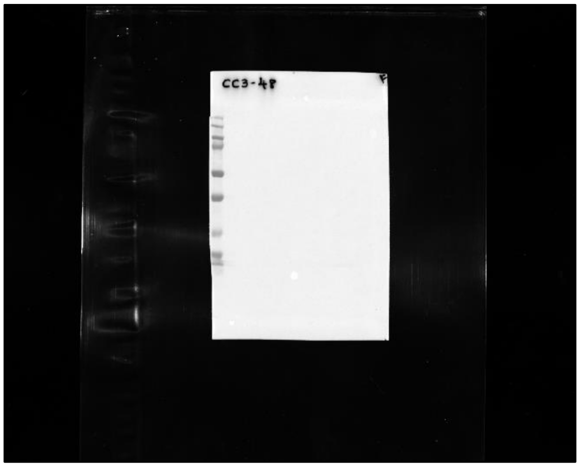

Caspase 8 protein after 24 & 48-hour treatment with ellagic acid

Developed blot (24 Hour)

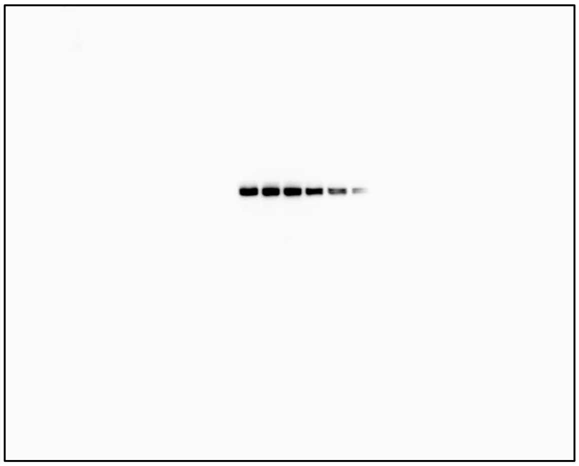

Colorimetric blot (24 Hour)

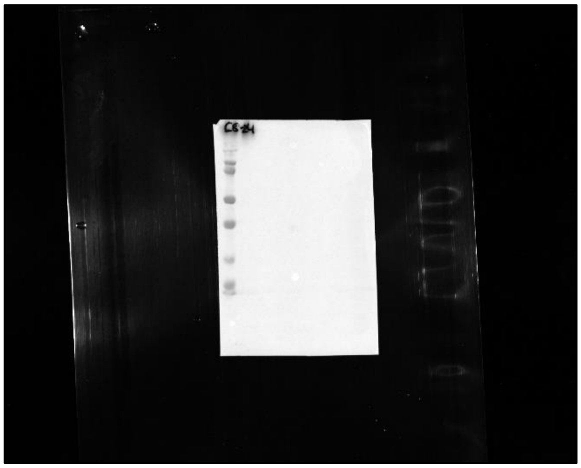

Developed blot (48 Hour)

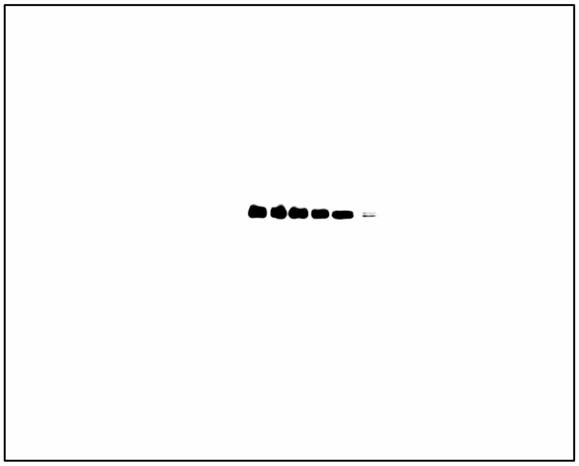

Colorimetric blot (48 Hour)

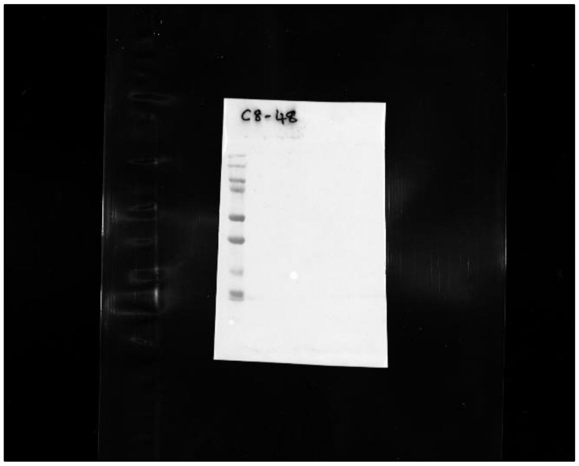

Cleaved caspase 8 protein after 24 & 48-hour treatment with ellagic acid

Developed blot (24 Hour)

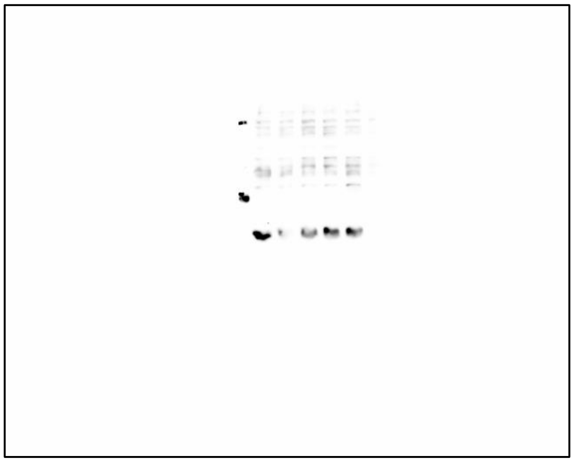

Colorimetric blot (24 Hour)

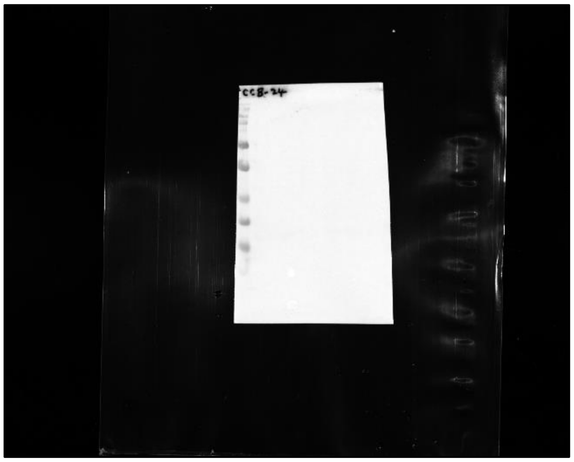

Developed blot (48 Hour)

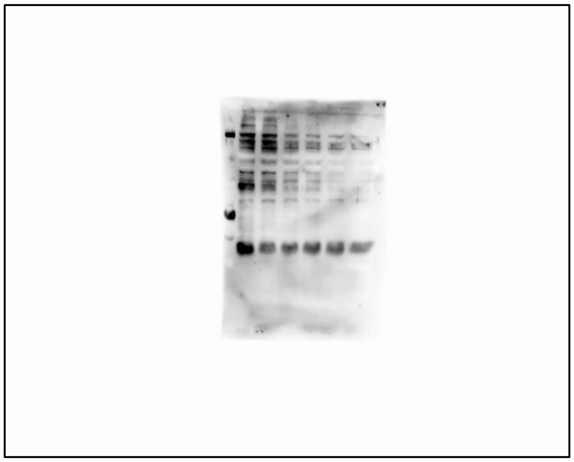

Colorimetric blot (48 Hour)

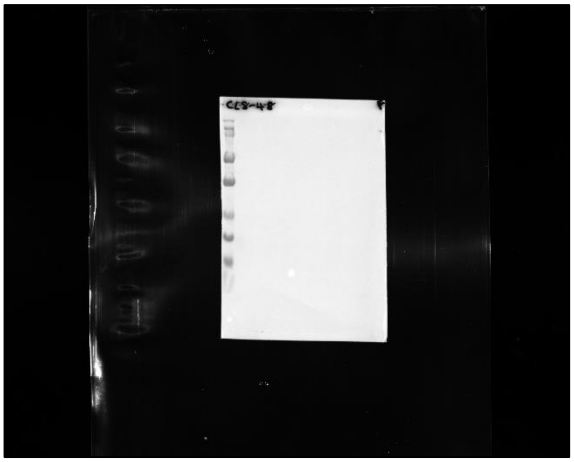

Cytochrome C protein after 24 & 48-hour treatment with ellagic acid

Developed blot (24 Hour)

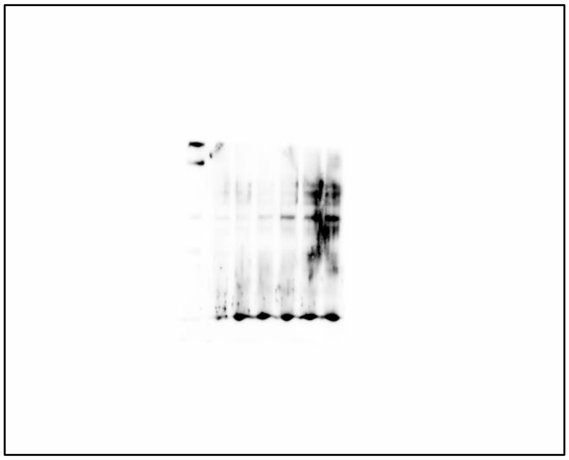

Colorimetric blot (24 Hour)

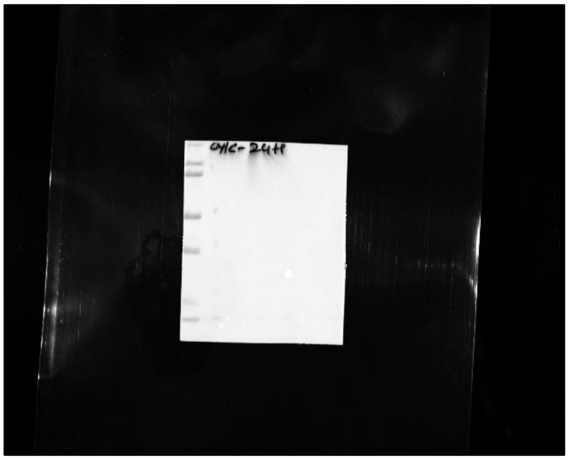

Developed blot (24 Hour)

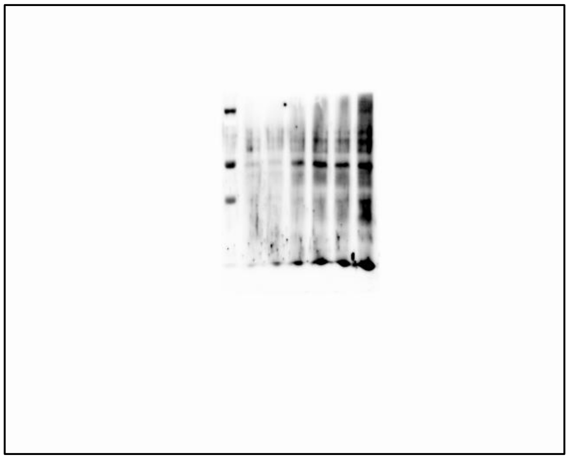

Colorimetric blot (24 Hour)

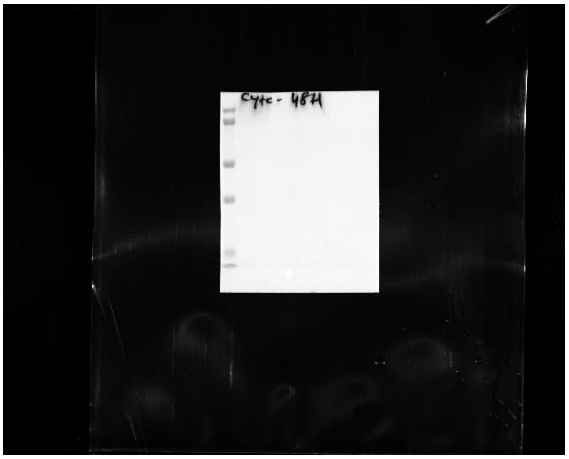

PARP-1 and Cleaved PARP-1 protein after 24 & 48-hour treatment with ellagic acid

Developed blot (24 Hour)	Colorimetric blot (24 Hour)
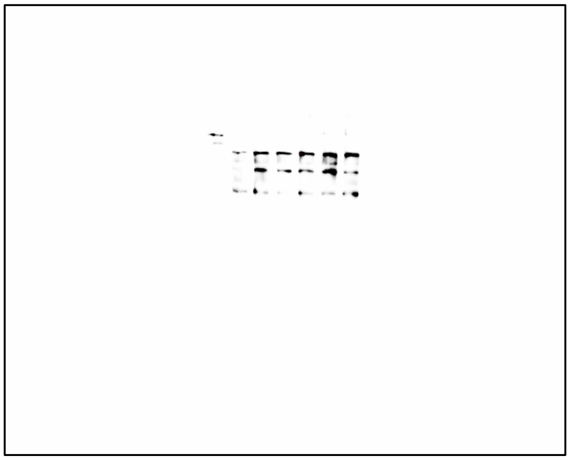	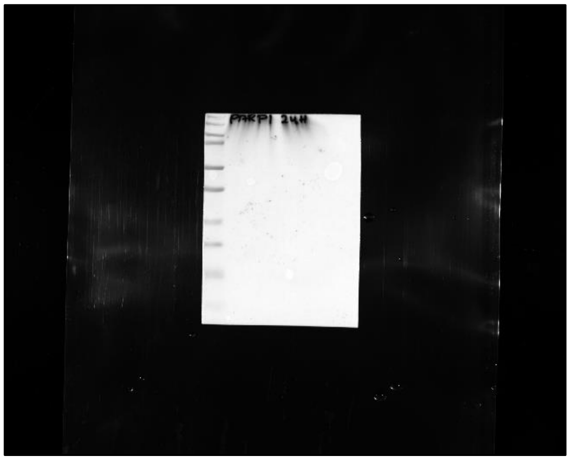
**Developed blot (48 Hour)**	**Colorimetric blot (48 Hour)**
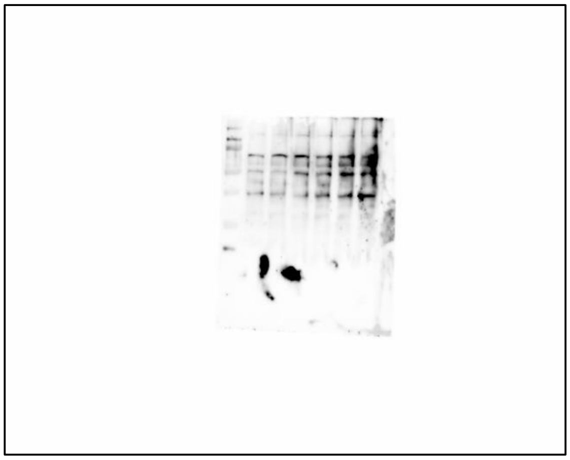	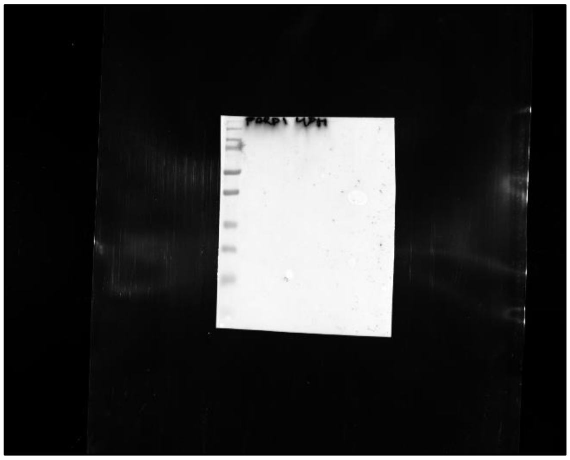

MLKL protein after 24 & 48-hour treatment with ellagic acid

Developed blot (24 Hour)

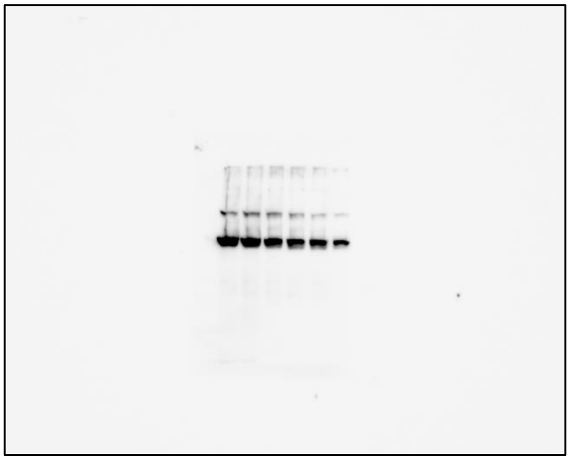

Colorimetric blot (24 Hour)

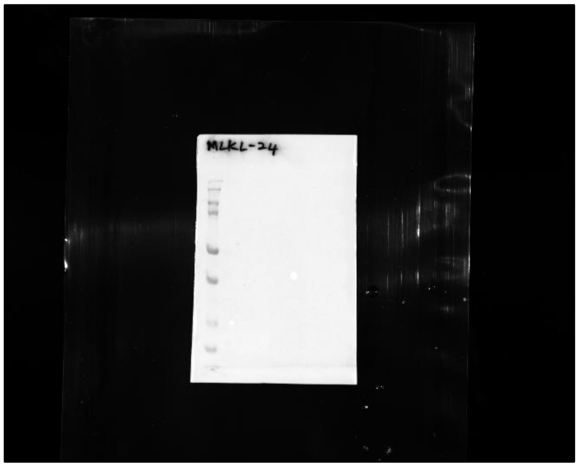

Developed blot (48 Hour)

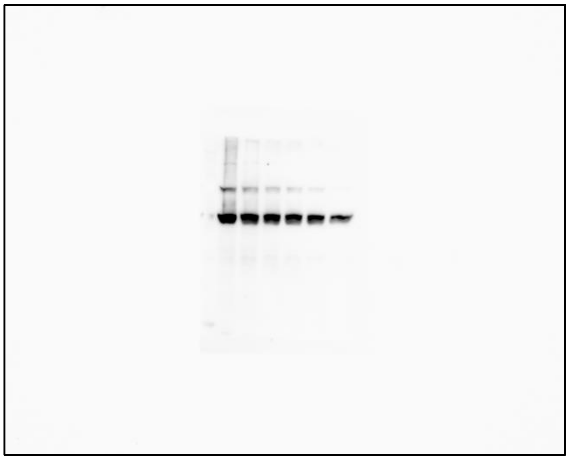

Colorimetric blot (48 Hour)

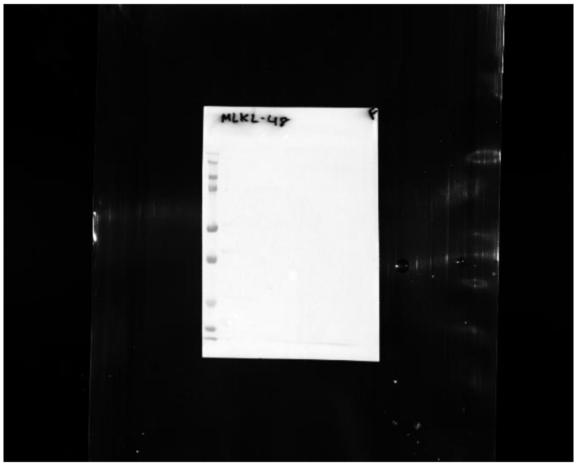

p-MLKL protein after 24 & 48-hour treatment with ellagic acid

Developed blot (24 Hour)	Colorimetric blot (24 Hour)
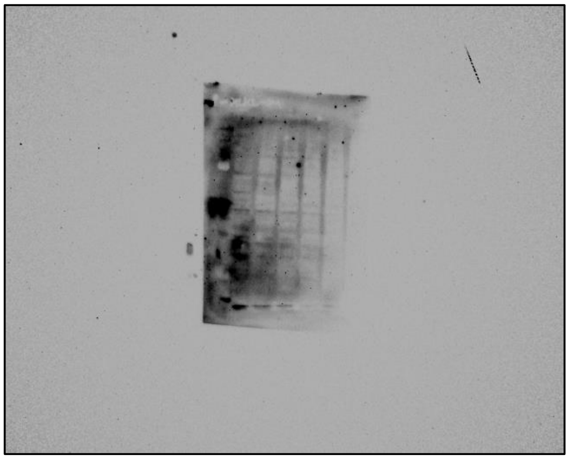	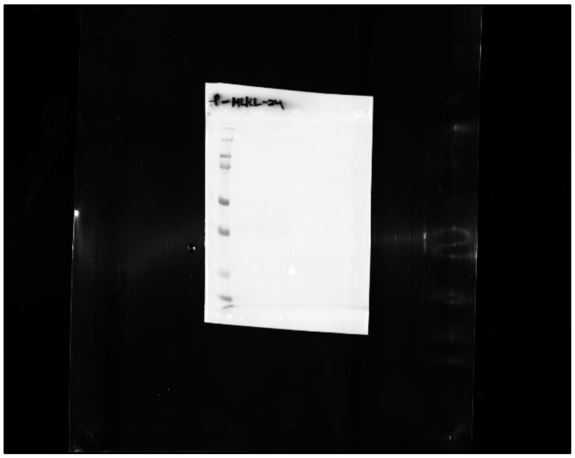

Developed blot (48 Hour)	Colorimetric blot (48 Hour)
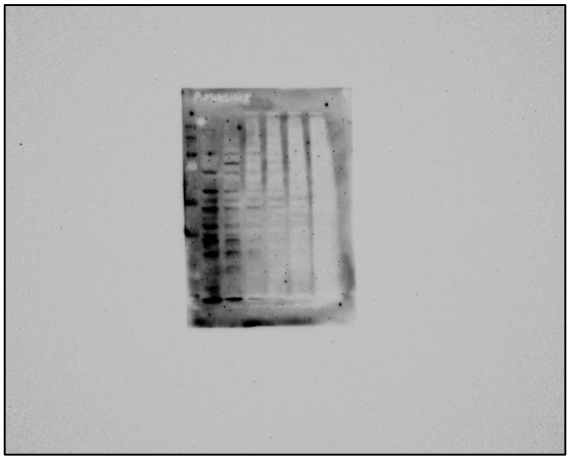	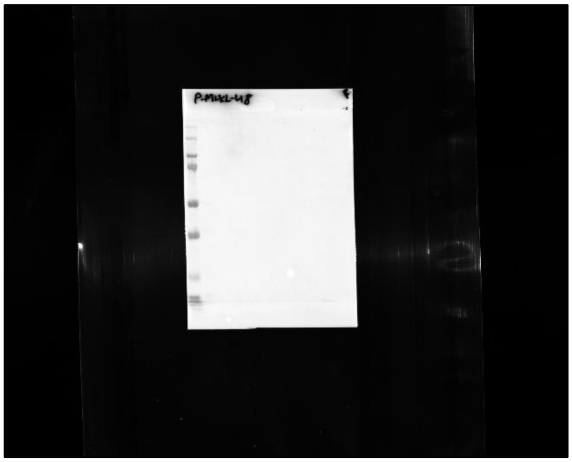

RIP protein after 24 & 48-hour treatment with ellagic acid

Developed blot (24 Hour)

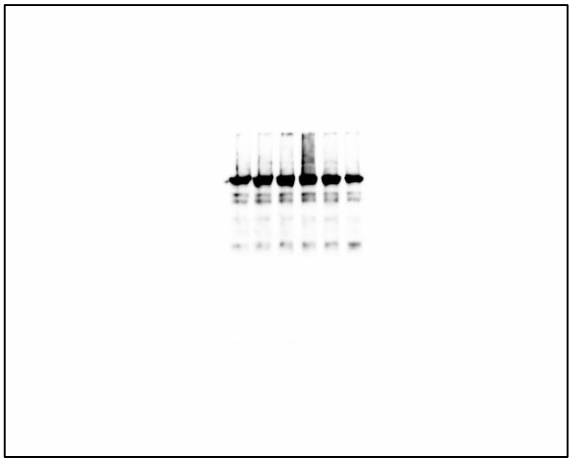

Colorimetric blot (24 Hour)

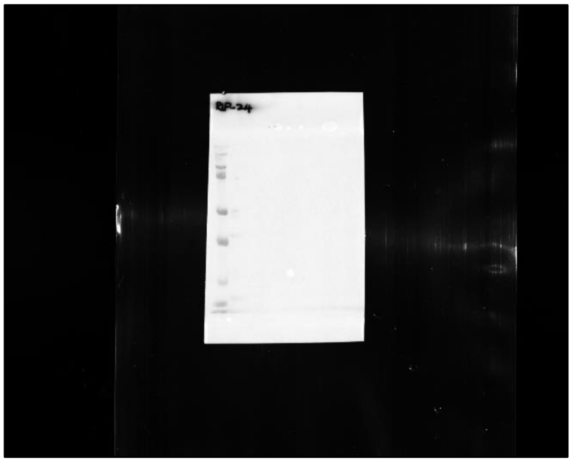

Developed blot (48 Hour)

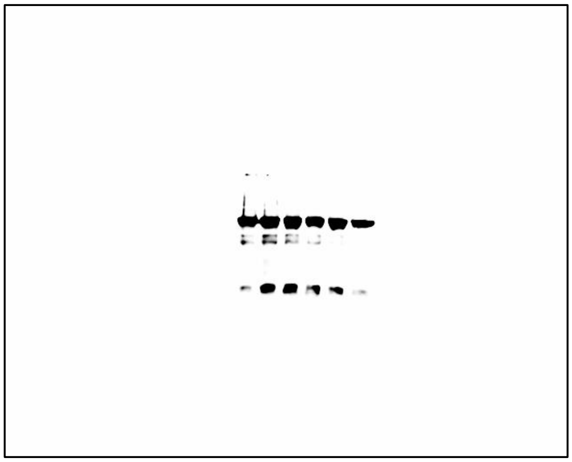

Colorimetric blot (48 Hour)

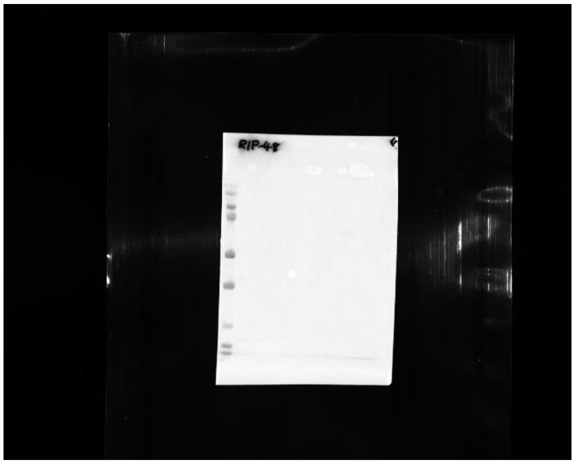

p-RIP protein after 24 & 48-hour treatment with ellagic acid

Developed blot (24 Hour)

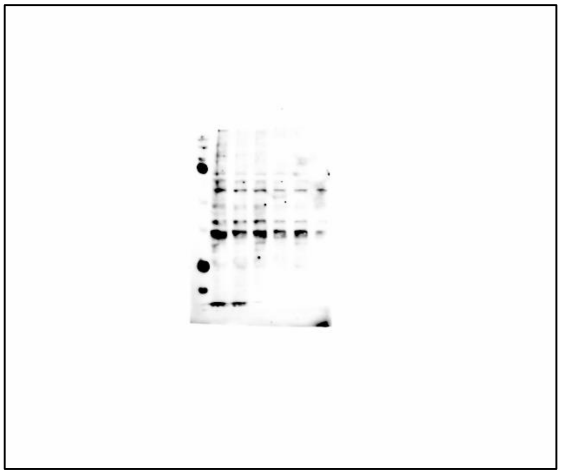

Colorimetric blot (24 Hour)

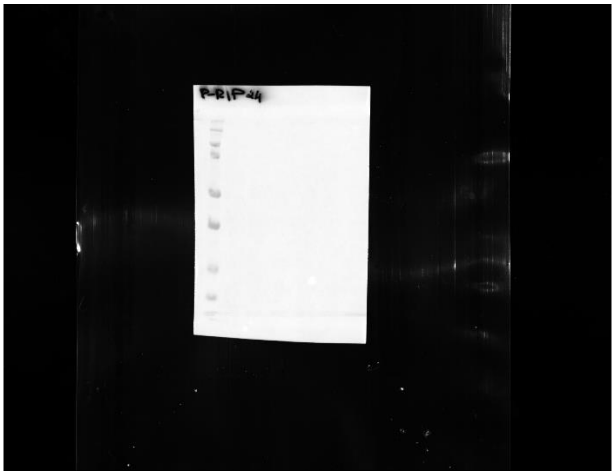

Developed blot (48 Hour)

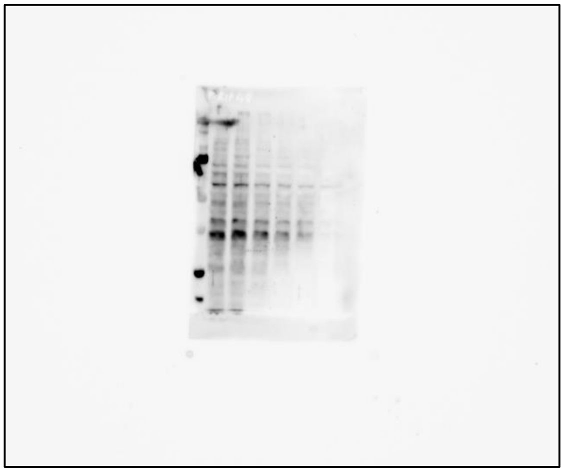

Colorimetric blot (48 Hour)

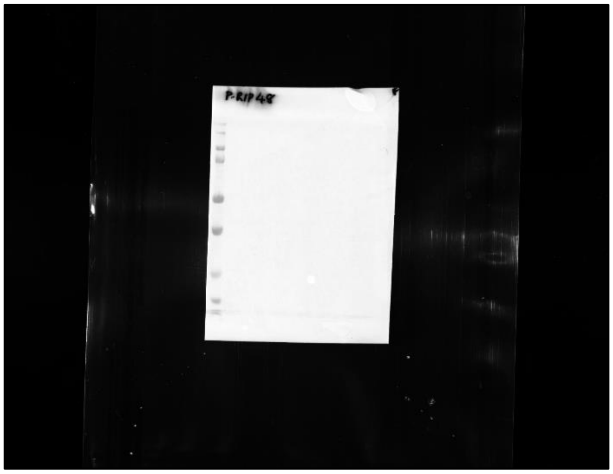

Cyclin D1 protein after 24 & 48-hour treatment with ellagic acid

Developed blot (24 Hour)

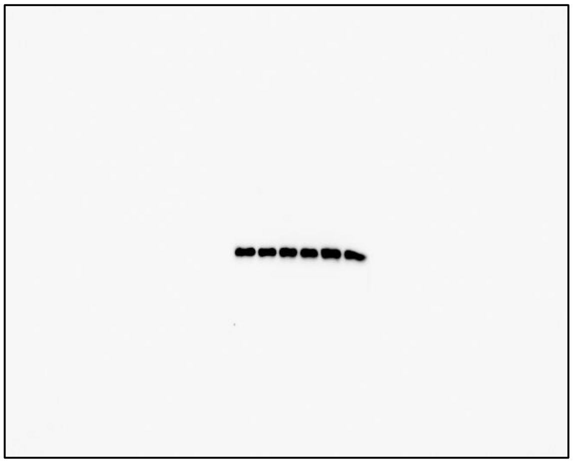

Colorimetric blot (24 Hour)

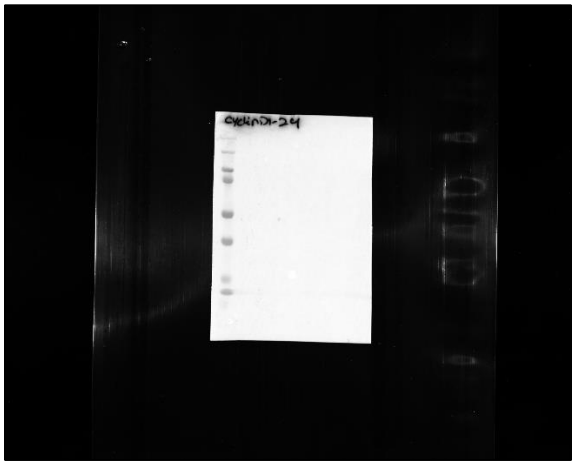

Developed blot (48 Hour)

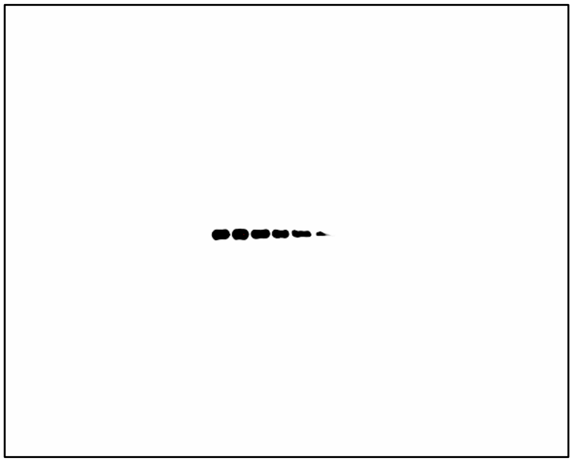

Colorimetric blot (48 Hour)

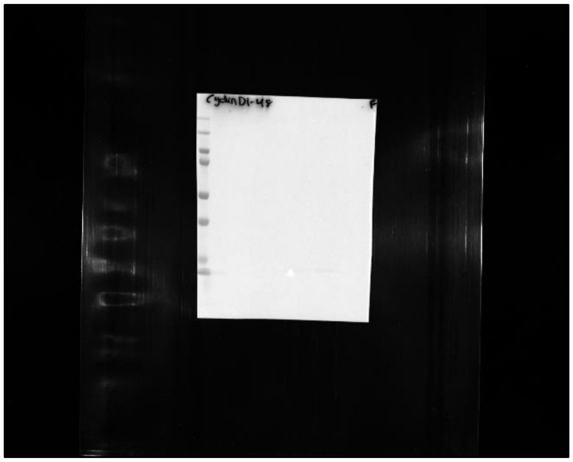

Cyclin A2 protein after 24 & 48-hour treatment with ellagic acid

Developed blot (24 Hour)

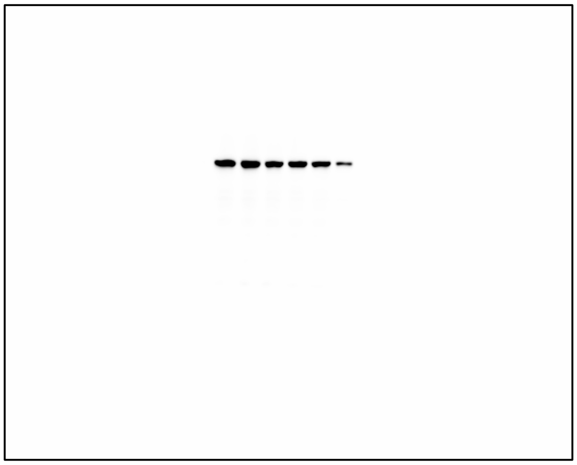

Colorimetric blot (24 Hour)

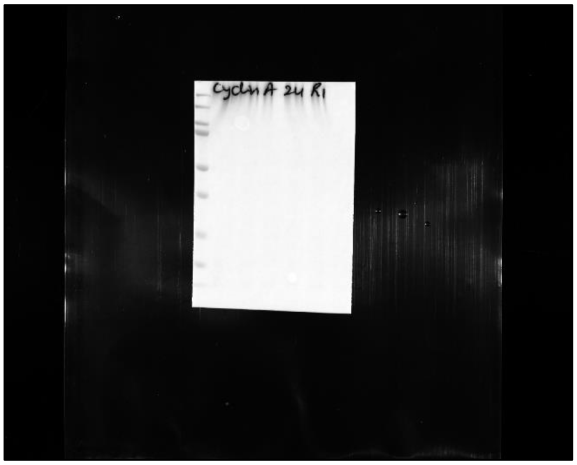

Developed blot (48 Hour)

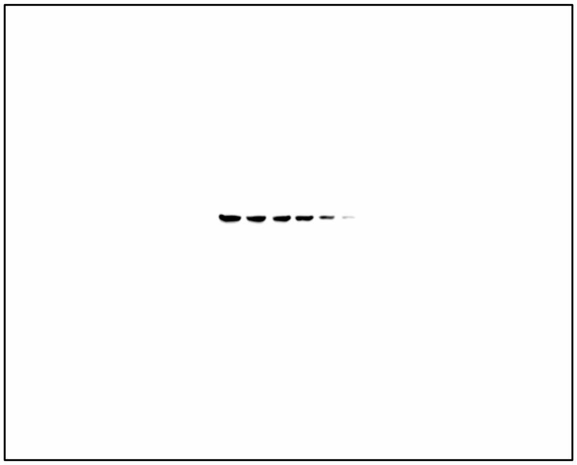

Colorimetric blot (48 Hour)

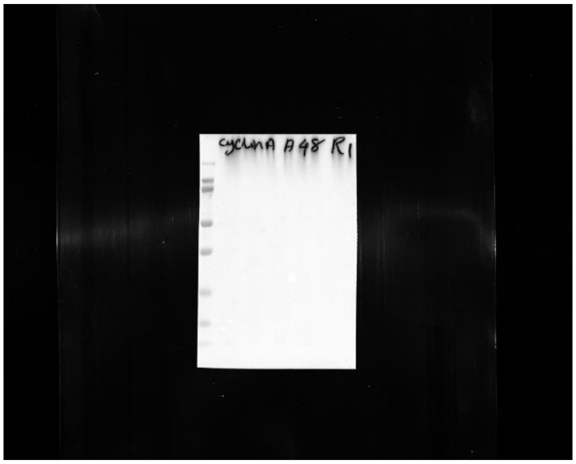

GAPDH protein after 24 & 48-hour treatment with ellagic acid

Developed blot (24 Hour)

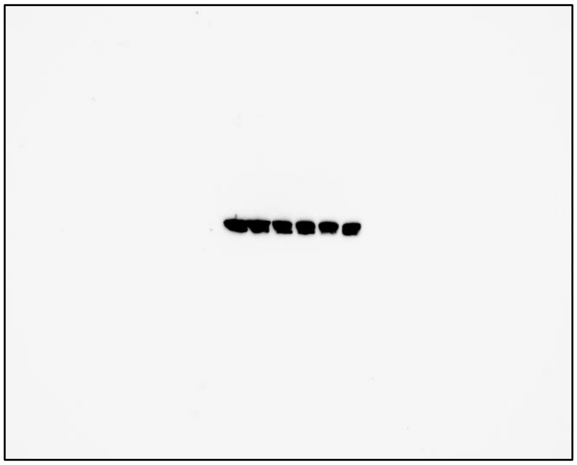

Colorimetric blot (24 Hour)

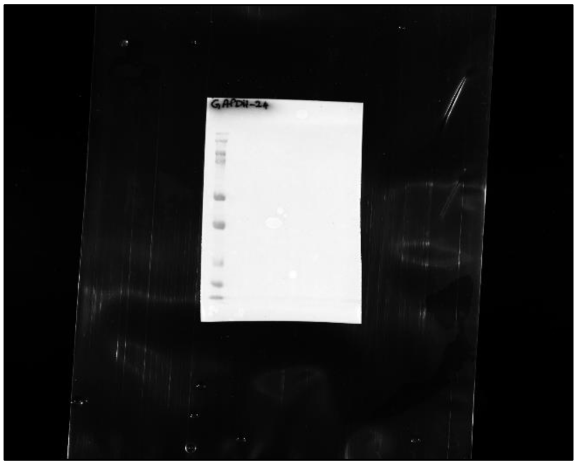

Developed blot (48 Hour)

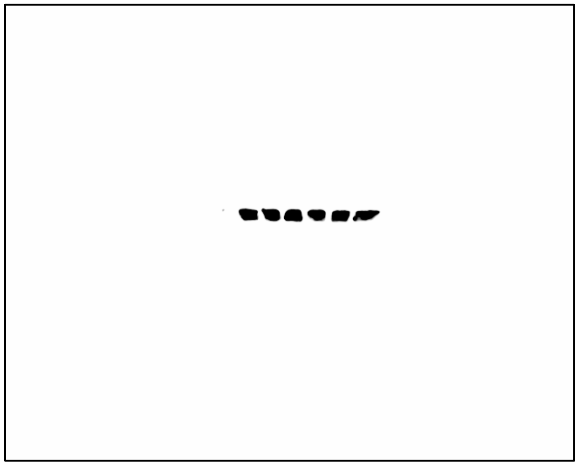

Colorimetric blot (48 Hour)

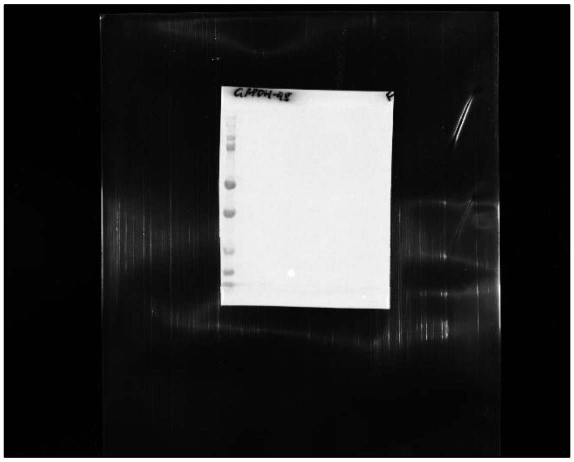

Supplement: S1 Raw images — (PDF) [file pone.0286274.s004.pdf]
