# Supplementary material for: Extraction, phytochemical characterization and anti-cancer mechanism of Haritaki churna: An ayurvedic formulation
Source: PLoS One. 2023 May 31;18(5):e0286274. doi: 10.1371/journal.pone.0286274 (PMC10231837; doi:10.1371/journal.pone.0286274)
Supplement: Attachment — Submitted filename: Response to Reviewers.docx [file pone.0286274.s005.docx]

From	11.03.2023

Dr. Vishal Trivedi

Professor,

Department of Biosciences and Bioengineering

Indian Institute of Technology-Guwahati

Guwahati-Assam India-781039

To

The Editor,

Plos One

Dear Editor,

Thank you for giving us the opportunity to submit a revised draft of our manuscript titled **“Extraction, phytochemical characterization and anti-cancer mechanism of Haritaki Churna: An ayurvedic formulation” to PlosOne journal** We appreciate the time and work you and the reviewers put into giving useful criticism on the paper. We have been able to incorporate changes to reflect most of the suggestions provided by the reviewers. We have highlighted the changes within the manuscript.

The revised manuscript has sufficient merit to be published in your Journal. We have no conflict of interest in any part of this article and none of the material has been published or is under consideration elsewhere.

All authors have contributed to, seen, and approved the final, submitted version of the manuscript.

With best regards,

Vishal Trivedi

**Response to reviewer**

**Reviewer 1:**

**Comment 1:** The authors could improve the overall language of this manuscript to increase readability.

**Response:** English is edited, corrected by online English editing software Grammarly and another colleague proof read the manuscript. Language is of journal’s desired standard.

**Comment 2:** Introduction: The introduction section of this study fails to relate with the previous study accurately. Rewrite this section and relate why this study is novel and its importance.

**Response to comment 2:** The introduction section has been modified as per the requirement. (L56-L80)

**Comment 3:** Conclusion: This section is not properly written. You are suggested to write the major findings and its significance in few sentences.

**Response to comment 3:** As suggested, The conclusion section has been modified as per the requirement. (L626-639)

**Comment 4:** Some figures are blurry. Include figures with correct resolution

**Response to comment 4:** The figures re-submitted to journal are of publication quality with high resolution of 300 dpi. All the figures have been prepared again as per journal requirements.

**Reviewer 2:**

**Comment 1:** The chemical characterization has been done but, due to the lack of reference standards, the quantification of ellagic acid as well other effective compounds is missing.

**Response to comment 1:** Quantification of gallic acid, methyl gallate and ellagic was done using the respective standards. The standard curves were drawn after spiking different concentrations of GA, MG and EA in a gradient HPLC chromatogram. The calibration curve of different phytochemical was used to quantify. **This data is included in supplementary data-2.** The write up of quantification of GA, MG and EA are included in main manuscript from Line 477-481.

**Comment 2:** Authors should have completed the gastrointestinal stability simulation, by adding the simulation of the activity of bile salts and pancreatin at pH 6.8-7.2.

**Response to comment 2:**

- In order to test the efficacy of HC and HCAE in simulated intestinal fluids (SIF), two types of dissolution media were used that could mimic the condition in fasted (FaSSIF) and fed state (FeSSIF). These dissolution media primarily contain bile salts and pancreatin at pH 6.5 and pH 5.8
- The recipe to prepare FaSSIF and FeSSIF have been included in Materials and methods section as section **2.6.2** and **2.6.3**
- HC was extracted in FaSSIF and FeSSIF and no significant changes in the compositional make up of the extracts when compared to its aqueous extract. Further, HCAE was incubated in FaSSIF and FeSSIF from 0-6 hours at a time interval of 2 hours. A decrease in reduction in intensity of compound 11, 12 (chebulagic acid and chebulinic acid) and an increase in intensity of compound 13 (ellagic acid) was observed with progression of incubation periods. The increase in intensity of ellagic acid in HCAE incubated with FaSSIF and FeSSIF showed reduction in IC_50_ values when tested on DLD1 cancer cells for 48 hours of treatment.
- **The write up explaining the result have been discussed in section 3.7 from Line 424-440**
- **A new figure (Figure 7) have been included in manuscript to explain the results of effect of simulated intestinal conditions on HCAE**

**Comment 3:** Finally, molecular mechanism was not investigated.

**Response to comment 3:**

- Molecular mechanism explaining the death of colorectal cancer cells is discussed in section 3.11. Further, the in-depth mechanism of killing of DLD1 cancer cells upon treatment with ellagic acid was discussed with respect to up-regulation and down-regulation of apoptotic, necroptotic and cell signaling proteins through western blotting.
- **The write up of results of western blotting experiments are discussed in section 3.10 & 3.11 from Line 521-563**
- Two new panels (**9G** & **9H**) have been added in Figure **9**

**Comment 4:** Please check carefully spelling errors.

**Response to comment 4:** Spelling errors have been corrected wherever necessary. English is edited, corrected by online English editing software Grammarly and another colleague proof read the manuscript. Language is of journal’s desired standard.

**Reviewer 3:**

**Comment 1:** The pictures in the article are not clear and the pixels do not meet the publication requirements.

**Response to comment 1:** The figures re-submitted to journal are of publication quality with high resolution of 300 dpi. All the figures have been prepared again as per journal requirements

**Comment 2:** The purity of the compound is not high.

**Response:** I agree with your observation as few minor peaks are appearing in HPLC chromatogram of isolated phytochemicals from HCAE (Supplementary data 2). The most of the phytochemicals isolated from HCAE and used in the current study are more than 95% pure. It is known that the isolated pure phytochemicals from plant are prone to degradation, modification (oxidation) and appearance of additional peaks on the HPLC chromatogram could be due to these reasons. The compound present in minor peaks is very less and may not cause any significant impact on the anti-cancer activity reported by us.

**Comment 3:** Neither the fraction nor the compound activity is very strong.

**Response to comment 3:** The individual fractions anti-cancer activities has been discussed section **3.8**. All the fractions are not active, but fraction 2 and fraction 5 show greater activity than the aqueous extract. Since it was a deductive approach, we were able to rule out certain fractions that were not as good as fractions 2 and 5. Further, individual anti-cancer activities of each compound have also been reported in the manuscript which correlate with the previously published literature.

**Reviewer 4:**

**Comment 1:** In figure **4**, the retention time of B, and D need to be increased for easy observation about all compounds in the condition.

**Response to comment 1:** Figure **4** have been updated. The retentions times of all the fractions now show from 0 to 30 minutes

**Comment 2:** In figure 5, the structure of the compound is recommended to be beautified in the same format.

**Response to comment 2:** Figure **5** have been beautified as per requirement.

**Comment 3:** The accuracy of the mass spectrometry data in the article needs to be improved to four decimal places, and needs to be consistent, such as line 338 and 341.

**Response to comment 3:** The accuracy of the mass spectrometer data has been updated to four decimals in section **2.13** and section **3.6**

**Comment 4:** In 3.5, please add the amount of each fraction separated.

**Response to comment 4:** The amount of each fraction separated is added in section **3.5** from Line 350-352

**Reviewer 5:**

**Comment 1**: The manuscript needs to be polished the English grammar or spelling by a native speaker.
Inconsistent hyphenation throughout the manuscript. Like anti-cancer vs anticancer, gastro-intestinal vs gastrointestinal, etc. Both ways are acceptable, but you have to keep consistent.
American English and British English are mixed used.

**Response to comment 1**: English grammatical mistakes were corrected through out the manuscript and consistency of single language system is maintained.

**Comment 2**: The last decade has seen remarkable progress in cancer research, with a significant update to many theories. But most of the citations here have been published for more than ten years.

**Response to comment 2:** The citations with reference to cancer research have been updated wherever applicable. New references have been added and old references have been removed.

**Comment 3:** All experiment data need to have supported tables or figures, for section 3.1, no tables or figures are cited.

**Response to comment 3:** Table **1** has been cited in section **3.1** and has been updated in line 297

**Comment 4**: Section 3.3, the authors stated, “HC aqueous extract had not affected the cellular viability of HEK-293 cells up to concentration of 300 µg/ml. In addition, it is not affecting the cellular morphology as well (Figure 2B). However, we can see significant changes in morphology in HEK293 in Figure 2B. And there is no discussion for these changes.

**Response to comment 4**: “Blebbing in HEK-293 cells treated with higher concentration (500µg/ml) of HCAE was observed (Figure 2**B**). However, these protrusions have been found in HEK-293 cells at doses greater than those necessary to kill colorectal cancer cells”. The discussion pertaining to comment 4 have been updated in Line 328-330

**Comment 5:** Figure 5, since there are different subfigures, please cite them separately according to the results.

**Response to comment 5:** Subfigures of figure **5** have been cited next to the names of the compounds identified in section **3.6**

**Comment 6:** Section 3.9, “This increase in anticancer activity may be attributed to their antagonistic action. Their antagonistic action may be (a) because of these phytochemicals competing for the same protein which is crucial in pathway of killing cancer cells or (b) may be because of formation of a complex polyphenol mixture which are unable to perform their individual activities. References to support this discussion are missing

**Response to comment 6:** Relevant reference supporting the statement is been added in the revised manuscript.

**Comment 7**: Section 3.10. Three independent repeated experiments are needed to get the mean ± SD/ SEM and reliable results. While the results here seem only come from a one-off experiment.

**Response to comment 7:** Standard deviation values for the experiment discussed in section **3.10** have been updated in Line 524-526

**Comment 8**: Current data is not strong enough to make the conclusion that “ellagic acid is capable of arresting cell cycle progression in S-phase and induction of apoptosis in colorectal cancer cells” More experiment such as Western Blotting to check the cell cycle stage-dependent makers is needed, such as Cyclin A, Cyclin B…
The same for apoptosis, more experimental evidence is needed.

**Response to comment 8**:

- Molecular mechanism explaining the death of colorectal cancer cells is discussed in section 3.11. Further, the in-depth mechanism of killing of DLD1 cancer cells upon treatment with ellagic acid was discussed with respect to up-regulation and down-regulation of apoptotic, necroptotic and cell signaling proteins through western blotting.
- Western blotting of caspase 8, cleaved caspase 8, caspase 3, cleaved caspase 3, PARP-1, Bcl2, cytochrome-c, MLKL, p-MLKL, RIP, and p-RIP have been included in the results section which rule out extrinsic pathway of apoptosis and necroptosis happening upon treatment with ellagic acid at different concentration for 24 and 48 hours.
- Further, expression levels of cyclin A2 and cyclin D1 were recorded using western blotting. Cyclin A2 levels lowered in both time points, but cyclin D1 was predominantly lowered in 48 hours of treatment.
- The write up of results of western blotting experiments are discussed in section **3.10** & **3.11** from Line 521-563
- Two new panels (**9G** & **9H**) have been added in Figure **9** showing the western blot results obtained.
- The discussion and conclusion section have also been updated with reference to the new data included in the manuscript.
